# Total Synthesis of the Chlorinated Pentacyclic Indole Alkaloid (+)-Ambiguine G

**DOI:** 10.1021/jacs.1c05762

**Published:** 2021-07-19

**Authors:** Lingbowei Hu, Viresh H. Rawal

**Affiliations:** Department of Chemistry, University of Chicago, 5735 South Ellis Avenue, Chicago, Illinois 60637, United States

## Abstract

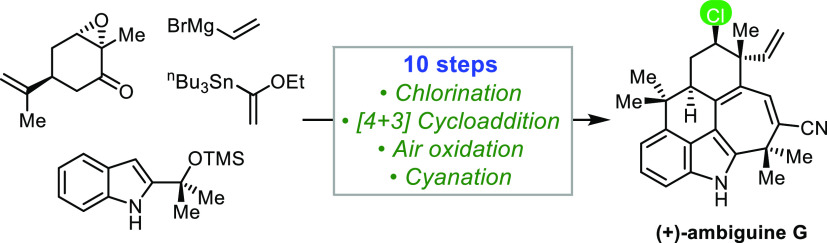

Reported herein is the total synthesis of (+)-ambiguine G, the first member of the chlorinated pentacyclic ambiguines to yield to chemical synthesis. The synthesis is accomplished through a convergent strategy that proceeds in 10 steps from (*S*)-carvone oxide. Pivotal to the concise route is the successful realization of a [4+3] cycloaddition that conjoins two easily synthesized components of the carbon framework of the natural product. Also featured in the synthesis is the efficient, diastereoselective construction of a key vinylated chloro ketone and the unprecedented, one-pot reduction–elimination–oxidation sequence that transforms an enone to an advanced hydroxylated-diene intermediate.

The ambiguines are a subset of the large hapalindole family of more than 80 cyanobacteria metabolites that also includes the fischerindoles and welwitindolinones.^1,2^ The first of the ambiguines were identified by Smitka and Moore in 1992 while screening fungicidal extracts primarily from the terrestrial cyanophytes *Fischerella ambigua*.^3^ Although the full bioactivity profiles of these alkaloids have yet to be fully assessed, several members have displayed useful properties. Of note, ambiguine I isonitrile (**4**) is not only a stronger antibacterial and antifungal agent than established clinical agents but also a potent NF-κB inhibitor (IC_50_ = 30 nM), with cytotoxic activity against HT-29 colon cancer and MCF-7 breast cancer cells (Figure 1).^4,5^ Structurally, all ambiguines contain the tetracyclic core of the hapalindoles, but 13 of the 18 members possess an additional, seven-membered ring that connects the indole to the distal six-membered ring. Furthermore, over half of the ambiguines possess a chlorine atom at C13, rendering them significantly more difficult as targets for synthesis.^1,6^ The intricate polycyclic architecture and the unpredictable reactivity of the pentacyclic ambiguines present a significant challenge to the state-of-the-art of synthesis, one that went unmet despite numerous efforts over many years.^7,8^ It was only in 2019 that the first pentacyclic member of this family of natural products succumbed to synthesis. Two contemporaneous publications, one by Sarpong and co-workers and the other by us, presented distinctly different strategies for the synthesis of ambiguine P (**7**).^9^ We now report the total synthesis of (+)-ambiguine G (**8**), the first member of the chlorinated pentacyclic ambiguines to yield to chemical synthesis.^10^

**Figure 1:**
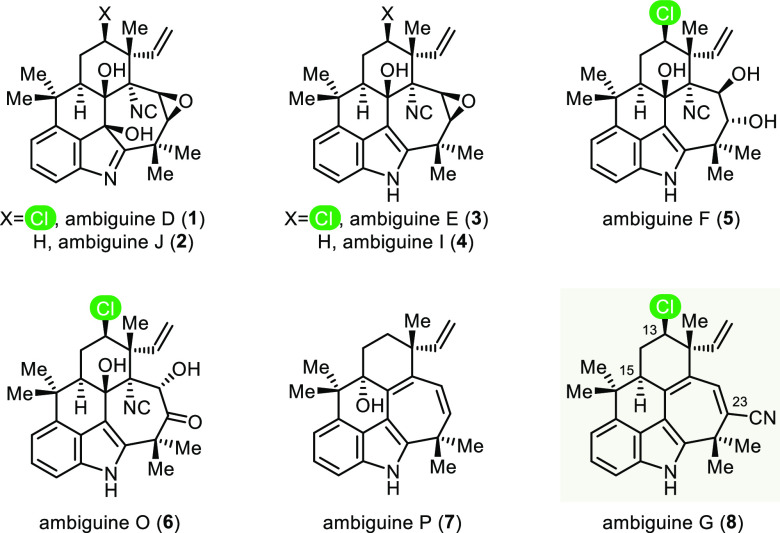
Selected members of the ambiguine natural products.

Our strategy to ambiguine G (**8**) is intimated in the retrosynthesis shown in Scheme 1 and is enabled by three key insights. First, the chlorine atom at C13 would be installed early in the synthesis to avoid potential rearrangements induced by the adjacent vinyl group in a rigid, advanced intermediate, as observed in the welwitindolinones.^11^ Second, through advanced model studies, we determined that the desired [4+3] cycloaddition reaction, which was unrealized in our previous ambiguine synthesis,^9b^ could be rendered efficacious by using an alkoxy diene instead of a siloxyl diene. Lastly, a removable functionality at C15 with low tendency to leave as a cation was deemed essential for the late-stage functionalization of C23. Otherwise, installation of the nitrile group at that position, whether through site-selective, direct electrophilic cyanation or via halogenation followed by transition metal catalyzed coupling with cyanide, was expected to be complicated by untoward reactions (e.g., proton loss from C15).

**Scheme 1:**
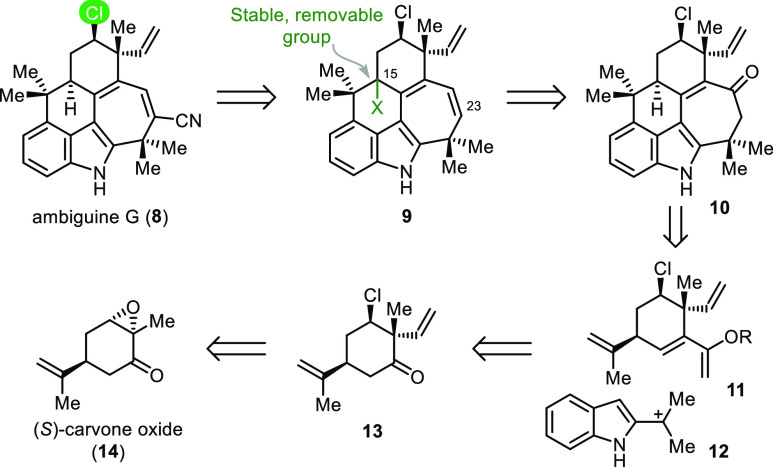
Retrosynthetic Analysis

Our synthesis of ambiguine G (**8**) commenced with the preparation of chloro ketone **17**, a functionalized six-membered ring unit common to numerous members of the hapalindole family. While seemingly simple, ketone **17** presents unique challenges, and the only reported synthesis of it requires 10 steps.^12^ In devising an alternate route to **17**, we planned to install the chloride via a stereoinvertive-deoxychlorination of hydroxy ketone precursor **16**, which mapped nicely over (*S*)-carvone oxide, provided a vinyl group could be introduced from the side opposite that of the isopropenyl unit. In simple carvone derivatives, however, it is well documented that carbon electrophiles are introduced at C2 cis to the isopropenyl unit due to stereoelectronic factors. Therefore, installation of a substituent trans to the isopropenyl unit would require harnessing the chirality of a preexisting functionality on a carvone derivative, thereby overriding the intrinsic diastereoselectivity. With this recognition, we examined different strategies with the goal of preinstalling a hydroxyl group at C3 and using it to direct a vinylation reaction. Success was achieved through the method reported by Coltart and co-workers.^13^ Addition of vinylmagnesium bromide to tosylhydrazone **15**, which was easily prepared from commercially available (*S*)-carvone oxide,^14^ followed directly by copper(II) chloride mediated hydrolysis of the hydrazone provided ketone **16** with nearly complete diastereoselectivity (Scheme 2).^15^ The vinyl addition took place as desired from the side away from the isopropenyl unit, ostensibly directed by coordination of the Grignard reagent with the alkoxide intermediate.^13a−13c^ To our knowledge, this epoxyhydrazone-mediated directed introduction of a carbon substituent α to a carbonyl group has not been utilized in natural product synthesis.^16^ Conversion of the vinyl-alcohol product **16** to chloro ketone **17** was accomplished using *N*-chlorosuccinimide and PPh_3_ with complete stereoinversion at the chlorine attaching carbon. The stereoretentive chlorination product was not observed.

**Scheme 2:**
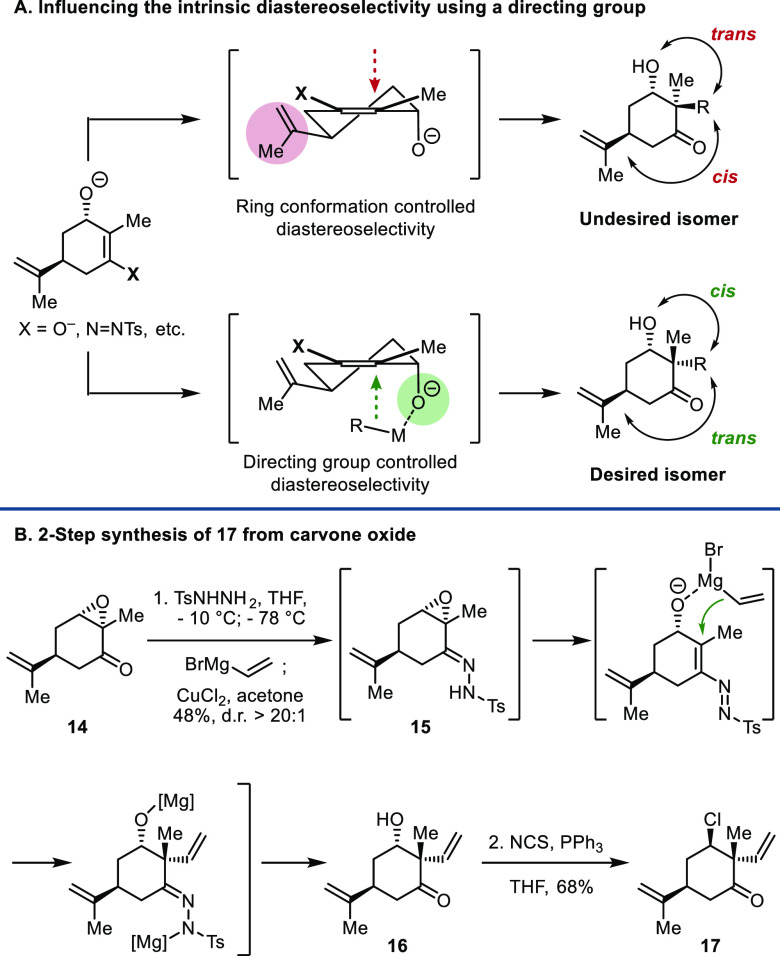
Synthesis of Chloro Ketone **17**

With a practical, two-step synthesis of chloro ketone **17** in hand, we focused our attention on assembling the carbon framework of the natural product via the [4+3] cycloaddition reaction.^17^ Although our published route to ambiguine P (**7**) was inspired by this cycloaddition as the key step, in practice it proved unsuccessful.^9b^ Rather than forging two C–C bonds to form the seven-membered ring, the reaction gave what is effectively the Friedel–Crafts alkylation product of the silyl enol ether and the benzylic cation (cf. **11** + **12**, Scheme 1). We reasoned that the reaction may proceed in a stepwise manner, wherein the labile silyl group falls off after formation of the first C–C bond to give an “interrupted” [4+3] product. On the basis of this hypothesis, we examined the cycloaddition reaction of ethoxy diene **18**, which was easily synthesized from ketone **17** via triflation followed by Stille cross-coupling. To our delight, treatment of diene **18** and indolic silyl ether **19**(18) with TMSOTf promoted the desired [4+3] reaction to afford tetracycle **20** cleanly, with no evidence of the Friedel–Crafts reaction product (Scheme 3).

**Scheme 3:**
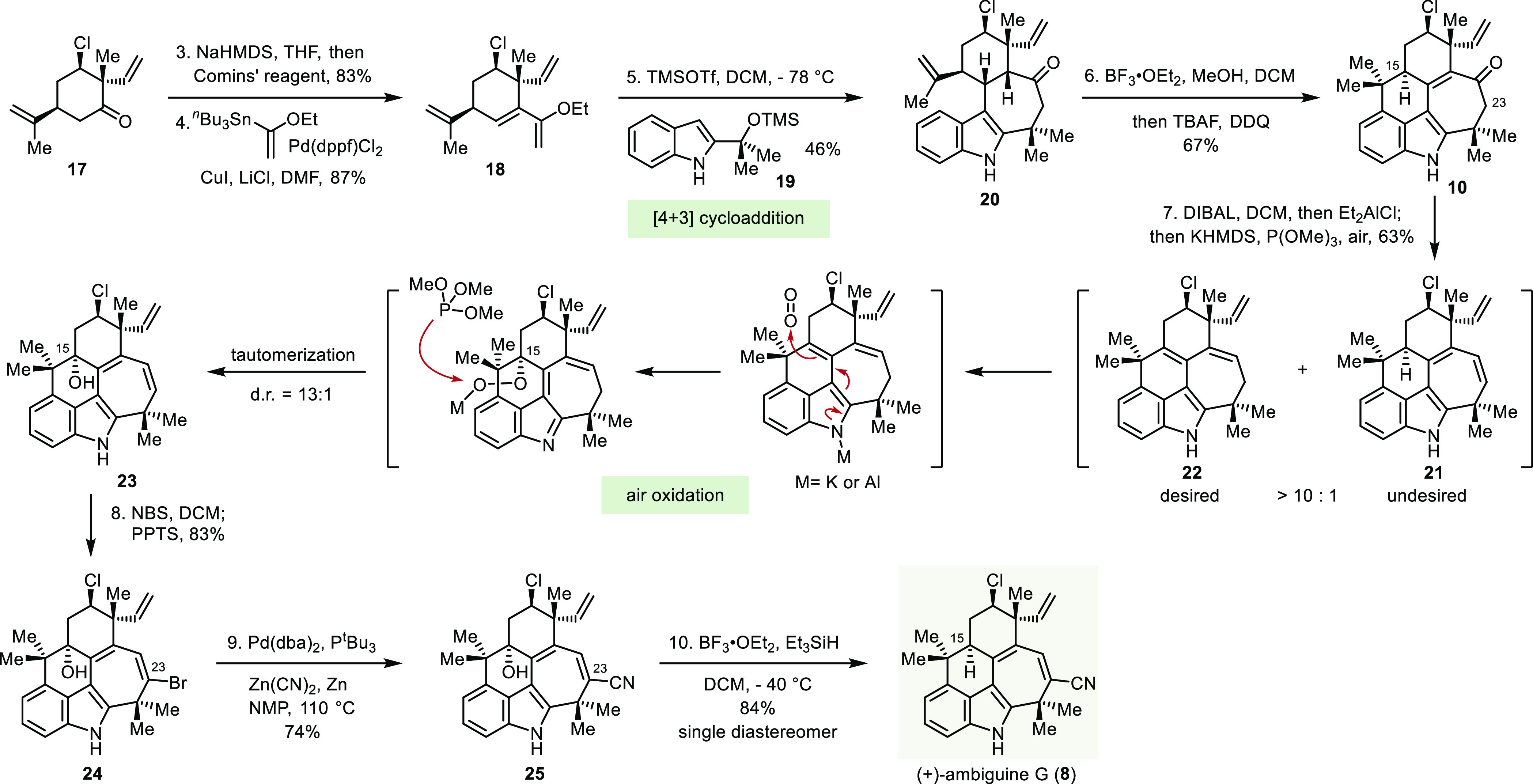
Total Synthesis of (+)-Ambiguine G

An efficient, two-pot reaction sequence was developed to transform tetracycle **20** to pentacyclic alcohol **23**. First, the [4+3] cycloadduct was treated with BF_3_·OEt_2_ to annulate the final ring through a Friedel–Crafts reaction. Subsequent addition of TBAF to quench the Lewis acid followed by DDQ oxidized the intermediate ketone to enone **10** in good yield. In the next protocol, DIBAL reduction of the carbonyl group and elimination of the resulting alkoxide using Et_2_AlCl produced a mixture of the conjugated diene **21** and the cross-conjugated diene **22**, greatly favoring the latter. The high regioselectivity for diene **22** likely reflects the stereoelectronic preference for elimination of the axially oriented C15 proton over the C23 proton in the conformationally rigid pentacyclic framework. The facile deprotonation at C15 also complicates the required electrophilic functionalization at C23 on diene **21** and necessitates the installation of a blocking group at C15. Fortunately, the C15 carbon of diene **22** was found to be unexpectedly electron rich, making it susceptible to air oxidation. On the basis of this realization, we developed a highly efficient procedure wherein after DIBAL reduction and Et_2_AlCl-mediated elimination, KHMDS was added to deprotonate the indole nitrogen and then the reaction mixture was exposed to air. Gratifyingly, the intermediate indole anion reacted with oxygen at C15, and the resulting hydroperoxide was reduced by the P(OMe)_3_ present to provide alcohol **23** in good yield and excellent diastereoselectivity (13:1).

Having installed the hydroxyl group at C15, and thereby forestalled side reactions arising from proton loss from that position, the next task was to introduce the nitrile group at C23. Although methods for the direct introduction of the nitrile group proved unsuccessful, treatment of diene **23** with *N*-bromosuccinimide selectively brominated the distal carbon of the conjugated diene without touching the vinyl group. A subsequent tautomerization in the presence of pyridinium *p*-toluenesulfonate produced alkenyl bromide **24** in high yield. The nitrile group was then introduced in good yield by a palladium-catalyzed coupling reaction.^19^ Having served its function, the hydroxyl group was removed under ionic hydrogenation conditions (BF_3_·OEt_2_ and Et_3_SiH) to afford (+)-ambiguine G (**8**), which was formed as a single diastereomer.

In summary, we have completed the enantiospecific synthesis of (+)-ambiguine G (**8**), a chlorinated member of the ambiguine family of indole alkaloids. The synthesis is accomplished through a convergent strategy that proceeds in 10 synthetic operations from (*S*)-carvone oxide and demonstrates (1) the construction of a key chlorine-substituted cyclohexanone precursor through an alkoxide-directed vinylation reaction, (2) the rapid assembly of the core skeleton of the natural product by a [4+3] cycloaddition reaction, and (3) the unprecedented, one-pot reduction–elimination–oxidation sequence that transforms an enone intermediate to a pivotal hydroxy diene. The efficiency of the route is expected to provide ready access to more intricate members of the pentacyclic ambiguines, as well as their analogues.

## Supporting Information Available

Experimental procedures and spectroscopic data for all new compounds (PDF)
