# Supplementary material for: Total Synthesis of the Chlorinated Pentacyclic Indole Alkaloid (+)-Ambiguine G
Source: J Am Chem Soc. 2021 Jul 19;143(29):10872–5. doi: 10.1021/jacs.1c05762 (PMC8509925; doi:10.1021/jacs.1c05762)
Supplement: Supplementary file 1 — ja1c05762_si_001.pdf [file ja1c05762_si_001.pdf]

Supporting Information

Total Synthesis of the Chlorinated Pentacyclic Indole Alkaloid (+)-Ambiguine G

Lingbowei Hu and Viresh H. Rawal*

Department of Chemistry, University of Chicago, 5735 South Ellis Avenue, Chicago, Illinois 60637, United States

Table of Contents

I. General Information 2

II. Synthetic Procedures towards Ambiguine G...2

III. References...13

IV. NMR Spectra..14

I. General Information

Unless stated otherwise, room temperature refers to 22-26 °C. Higher than room temperatures were maintained using pre-heated oil baths. Lower temperatures were maintained using acetone/CO_2(s) (−78 °C), MeCN/CO_2(s) (−40 °C) and water/ice (0 °C) baths. Dichloromethane (CH_2Cl_2 or DCM), tetrahydrofuran (THF), and dimethylformamide (DMF) were dried by passage through an activated alumina column purification system (Innovative Technology Inc. Pure SolvTM). All commercial reagents and solvents were used as received, except N-bromosuccinimide, (recrystallized from water) and N-chlorosuccinimide (recrystallized from acetic acid).

Thin-layer chromatography (TLC) was performed using EMD Millipore silica gel 60 Å F254 plates (250 μm) with F-254 fluorescent indicator and visualized by UV fluorescence quenching, ceric ammonium molybdate, or potassium permanganate staining. SiliCycle SiliaFlash P60 silica gel (particle size 40–63 μm) was used for flash chromatography. NMR spectra were measured on Bruker DRX and DMX spectrometers at 500 MHz for ^{1}H spectra and 126 MHz for ^{13}C spectra, respectively, and calibrated to either TMS (δ = 0 for ^{1}H), residual $CHCl_3$ (δ = 7.26 for ^{1}H and δ = 77.23 for ^{13}C), or residual benzene (δ = 7.16 for ^{1}H and δ = 128.06 for ^{13}C). Splitting patterns are reported as apparent. Mass spectral data was measured on Agilent technologies 6224 TOF LC/MS. Optical rotations were measured on a Jasco DIP-1000 polarimeter using a 100 mm path-length cell, c = g/100 mL. IR spectra were recorded on a Thermo Scientific Nicolet iS50 FT-IR spectrometer and are reported as frequency of absorption (cm^{-1}).

II. Synthetic Procedures towards Ambiguine G

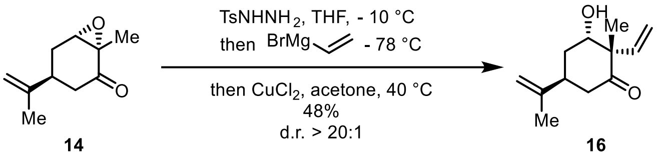

14 16

Hydroxy ketone 16. To a 500 mL round bottom flask was added (*S*)-carvone oxide (0.814 g, 4.90 mmol, 1 equiv.) and THF (8.2 ml, 0.6 M). The reaction mixture was then placed in a Cryocooler pre-set at - 10 °C. $TsNHNH_2$ (0.912 g, 4.90 mmol, 1 equiv.) was added in one portion and the resulting colorless solution was stirred at - 10 °C for 1.5 days, at which time the reaction turned slightly yellow. The flask was then cooled to – 78 °C and another 100 ml of THF was added. The reaction was stirred at this temperature for 5 minutes. Vinylmagnesium bromide (1 M in THF, 22 mL, 22 mmol, 4.5 equiv.) was added slowly into the reaction mixture over 20 minutes. The resulting deep brown solution was stirred at – 78 °C for another 1.5 hours, before the addition of 1 M HCl aq. (22 mL) The cooling bath was removed and acetone (100 ml) was added, followed addition of $CuCl_2$ (0.658 g, 4.90 mmol, 1 equiv.) in one portion. The copper salt soon dissolved upon heating to 40 °C, giving a dark brown solution, which gradually faded into a light brown solution over 1.5 hours. After a total of 2.5 hours at 40 °C (*Note 1*), the reaction was cooled down to room temperature. Most volatiles were removed *in vacuo* and the resulting black aqueous phase was extracted with EtOAc (1 x 50 mL, 3 x 20 mL). The organic layers were combined and washed with 1 M HCl (2 x 40 mL), brine (40 mL), dried over $MgSO_4$, filtered and concentrated *in vacuo*. The crude product was purified by flash chromatography (15% → 20% EtOAc/hexanes) to afford a mixture of the desired product and other inseparable by products. The mixture was then dissolved in hexane and ran

through a short celite plug to afford the desired product **16** (0.456 g, 48%) as a colorless oil.

Note 1: Long reaction time led to significant amounts of byproducts.

R_f= 0.27 (20% Et_2O/hexanes), visualized using $KMnO_4$

^{1}H NMR (500 MHz, $CDCl_3$): δ 6.48 (dd, J = 18.1, 11.1 Hz, 1H), 5.37 (dd, J = 11.2, 0.9 Hz, 1H), 5.23 (dd, J = 18.1, 0.9 Hz, 1H), 4.85-4.81 (m, 1H), 4.77-4.73 (m, 1H), 4.03 (app. t, J = 5.4 Hz, 1H), 2.87 (dq, J = 10.2, 5.4, 4.9 Hz, 1H), 2.59 (dd, J = 14.6, 10.3 Hz, 1H), 2.50 (ddd, J = 14.6, 5.3, 1.1 Hz, 1H), 2.10-2.03 (m, 2H), 1.97 (s, 1H), 1.78 (app. s, 3H), 1.30 (s, 3H).

^{13}C NMR (126 MHz, $CDCl_3$): δ 211.8, 147.1, 138.5, 116.4, 110.7, 75.8, 56.0, 42.3, 38.6, 32.4, 22.0, 21.2.

HRMS (ESI) *m/z* calc'd for $C_{12}H_{19}O_2$ $[M+H]^+$ 195.1385, found 195.1380.

$[\alpha]_D^{20}$= -11.7 ° (c=0.72, $CHCl_3$).

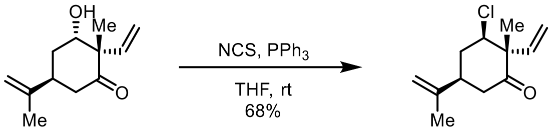

16 17

Chloroketone 17. To a flame-dried 25 mL round bottom flask was added N-chlorosuccinimide (116 mg, 0.87 mmol, 1.05 equiv.), THF (3.2 mL, 0.26 M) and PPh_3 (228 mg, 0.87 mmol, 1.05 equiv.) sequentially. The resulting pink slurry was stirred for 30 minutes at room temperature in the dark. Hydroxy ketone **16** (161 mg, 0.83 mmol, 1 equiv.) in 1.5 mL THF was then added to the reaction mixture, followed by another 1.7 mL of THF. The resulting slurry was stirred at room temperature for 17 hours, at which point the reaction turned into a burgundy solution. The reaction mixture was concentrated *in vacuo*. The resulting black oil was purified by flash chromatography (2% → 4% Et_2O/hexanes) to afford chloroketone **17** (120 mg, 68%) as a white solid.

R_f= 0.35 (10% Et_2O/hexanes), visualized using $KMnO_4$

^{1}H NMR (500 MHz, $CDCl_3$): δ 5.88 (dd, J = 17.5, 10.9 Hz, 1H), 5.36 (dd, J = 10.8, 0.7 Hz, 1H), 5.20 (dd, J = 17.5, 0.7 Hz, 1H), 4.86-4.81 (m, 1H), 4.78 (app. q, J = 1.0 Hz, 1H), 4.11 (dd, J = 12.1, 4.2 Hz, 1H), 2.62 (app. t, J = 13.9 Hz, 1H), 2.43-2.29 (m, 3H), 2.16 (app. q, J = 12.2 Hz, 1H), 1.76 (app. s, 3H), 1.39 (s, 3H).

^{13}C NMR (126 MHz, $CDCl_3$): δ 209.7, 145.6, 138.9, 116.8, 111.0, 64.2, 57.2, 42.2, 41.6, 36.8, 20.4, 16.1.

HRMS (ESI) *m/z* calc'd for $C_{12}H_{18}{}^{35}ClO$ $[M+H]^+$ 213.1046, found 213.1040.

$[\alpha]_D^{20}$= +4.5 ° (c=0.13, $CHCl_3$).

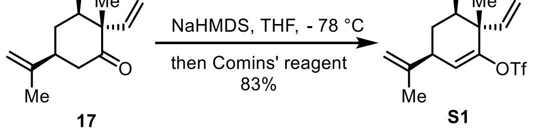

17 S1

Enol triflate S1. To a flame-dried 100 mL round-bottom flask was added chloroketone **17** (1.18 g, 5.55 mmol, 1 equiv.) and THF (18.5 mL, 0.30 M). The colorless solution was cooled to – 78 °C

followed by the slow addition of NaHMDS (1 M in THF, 6.38 mL, 6.38 mmol, 1.15 equiv.). The yellow solution was stirred at – 78 °C for 1.5 hour. Comins' reagent (3.27 g, 8.33 mmol, 1.5 equiv.) in THF (7.9 mL) was added dropwise (*Note 1*) and the reaction mixture was stirred at – 78 °C for another 1 hour (*Note 2*), before being quenched with saturated NH_4Cl aq. (20 mL). The reaction was warmed to room temperature, transferred into a separatory funnel containing 10 mL of H_2O and extracted with EtOAc (1 x 30 mL, 2 x 15 mL), washed with saturated NH_4Cl aq. (20 mL), brine (20 mL), dried over $MgSO_4$, filtered and concentrated *in vacuo*. The crude product was purified by flash chromatography (0% → 2% DCM/hexanes) to afford enol triflate **S1** (1.59 g, 83%) as a colorless oil.

Note 1: The addition of Comins' reagent needs to be slow to keep the temperature in the flask low to suppress chlorine elimination.

Note 2: Extended reaction time led to chlorine elimination.

R_f= 0.19 (1% Et_2O/hexanes), visualized using $KMnO_4$

^{1}H NMR (500 MHz, $CDCl_3$): δ 5.74-5.66 (m, 2H), 5.41 (d, *J* = 10.7 Hz, 1H), 5.31 (d, *J* = 17.3 Hz, 1H), 4.91-4.85 (m, 1H), 4.83 (dt, *J* = 1.4, 0.8 Hz, 1H), 4.10 (dd, *J* = 12.9, 3.3 Hz, 1H), 3.13 (ddd, *J* = 11.0, 6.0, 2.5 Hz, 1H), 2.28 (dddd, *J* = 13.3, 6.1, 3.3, 1.0 Hz, 1H), 1.95 (td, *J* = 13.1, 10.9 Hz, 1H), 1.75 (dd, *J* = 1.5, 0.8 Hz, 3H), 1.39 (s, 3H).

^{13}C NMR (126 MHz, $CDCl_3$): δ 150.9, 145.3, 138.7, 120.3, 118.9, 118.3 (q, $J_{19F\text{-}13C}$ = 319.8 Hz), 112.6, 63.4, 47.3, 42.4, 34.3, 20.2, 17.0.

HRMS (ESI) *m/z* calc'd for $C_{13}H_{17}{}^{35}ClF_3O_3S$ $[M+H]^+$ 345.0539, found 345.0530.

$[\alpha]_D^{21}$= -51.5 ° (c=0.25, $CHCl_3$).

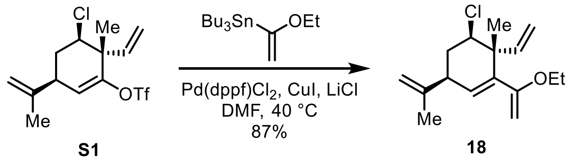

S1 **18**

Ethoxy diene 18. To a flame-dried 100 mL round-bottom flask was added enol triflate **S1** (1.587 g, 4.60 mmol, 1 equiv.) and DMF (bubbled with N_2 for 45 min before use, 35 mL). CuI (88 mg, 0.46 mmol, 0.1 equiv.), Pd(dppf)Cl_2·DCM (376 mg, 0.46 mmol, 0.1 equiv.), LiCl (1.034 g, 24.40 mmol, 5.3 equiv.) was added sequentially. Another 11 mL of DMF (bubbled with N_2 for 45 min before use) was added to make a 0.1 M solution. The reaction flask was purged with Ar for 10 min before the addition of tributyl(1-ethoxyvinyl)tin (1.79 mL, 5.29 mmol, 1.15 equiv.). The dark red solution was then heated to 40 °C. After 2.5 days at 40 °C, the brown solution was transferred into a separatory funnel containing NH_3·H_2O:H_2O 1:2 (90 mL), which was extracted with hexanes (1 x 90 mL, 3 x 40 mL). The organic phase was combined and washed with NH_3·H_2O:H_2O 1:2 (3 x 60 m), brine (60 mL), dried over Na_2SO_4, filtered and concentrated *in vacuo*. The crude material was purified by flash chromatography (basic alumina, pre-saturated with 10% NEt_3/hexanes for 30 minutes, flushed with 10% NEt_3/hexanes) to afford the ethoxy diene **18** as a colorless oil (1.068 g, 87%).

R_f= 0.31 (alumina, hexanes), visualized using $KMnO_4$

^{1}H NMR (500 MHz, benzene-d_6): δ 5.87 (dd, *J* = 2.7, 1.1 Hz, 1H), 5.82 (dd, *J* = 17.4, 10.7 Hz, 1H), 5.24-5.15 (m, 2H), 4.73 (dt, *J* = 1.8, 0.9 Hz, 1H), 4.69-4.66 (m, 1H), 4.12 (d, *J* = 1.7 Hz, 1H),

3.93 (dd, J = 12.5, 3.7 Hz, 1H), 3.85 (d, J = 1.6 Hz, 1H), 3.35 (ddq, J = 34.1, 9.4, 7.0 Hz, 2H), 2.63 (ddd, J = 11.0, 6.9, 2.6 Hz, 1H), 2.08 (dddd, J = 13.1, 6.9, 3.7, 1.1 Hz, 1H), 2.01 (td, J = 12.8, 10.9 Hz, 1H), 1.63 (s, 3H), 1.49 (dt, J = 1.8, 0.9 Hz, 3H), 1.03 (t, J = 7.0 Hz, 3H).

^{13}C NMR (126 MHz, benzene-d$_6$): δ 163.7, 147.1, 143.6, 142.8, 129.7, 114.5, 111.4, 84.6, 66.4, 63.1, 45.5, 44.9, 34.8, 20.0, 19.1, 14.5.

HRMS (ESI) *m/z* calc'd for $C_{16}H_{24}{}^{35}ClO$ [M+H]$^+$ 267.1516, found 267.1509.

$[\alpha]_D^{20}$= -96.5 ° (c=0.25, $CHCl_3$).

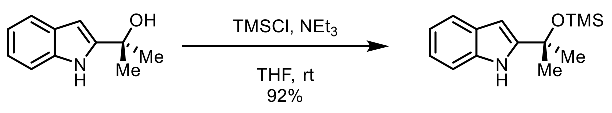

S2 19

TMS alcohol 19. To an oven-dried 100 mL round-bottom flask was added alcohol **S2**[1] (2.856 g, 16.3 mmol, 1 equiv.), NEt_3 (6.82 mL, 48.9 mmol, 3 equiv.) and THF (16.3 mL, 1 M). TMSCl (4.14 mL, 32.6 mmol, 2 equiv.) was added slowly at room temperature. The resulting solution was stirred at room temperature for 2 d before being quenched with saturated $NaHCO_3$ aq. (15 mL). The mixture was then transferred into a separatory funnel containing saturated $NaHCO_3$ aq. (75 mL) and H_2O (75 mL) and extracted with EtOAc (2 x 60 mL 1 x 20 mL). The organic phase was combined and washed with brine (50 mL), dried over Na_2SO_4, filtered and concentrated *in vacuo*. The crude product was purified by flash chromatography (3%→ 5% EtOAc/hexanes) to afford the desired TMS alcohol **19** as an off white solid (3.71 g, 92%).

R_f= 0.21 (5% EtOAc/hexanes)

^{1}H NMR (500 MHz, $CDCl_3$): δ 8.29 (s, 1H), 7.55 (dd, J = 7.8, 1.1 Hz, 1H), 7.36 (dd, J = 8.1, 0.9 Hz, 1H), 7.15 (ddd, J = 8.2, 7.1, 1.2 Hz, 1H), 7.07 (ddd, J = 8.0, 7.0, 1.0 Hz, 1H), 6.23 (dd, J = 2.1, 0.9 Hz, 1H), 1.67 (s, 6H), 0.10 (s, 9H).

^{13}C NMR (126 MHz, $CDCl_3$): δ 146.8, 135.3, 128.6, 121.5, 120.5, 119.7, 110.9, 96.5, 72.3, 31.7, 2.3.

HRMS (ESI) *m/z* calc'd for $C_{14}H_{22}NOSi$ [M+H]$^+$ 248.1471, found 248.1467.

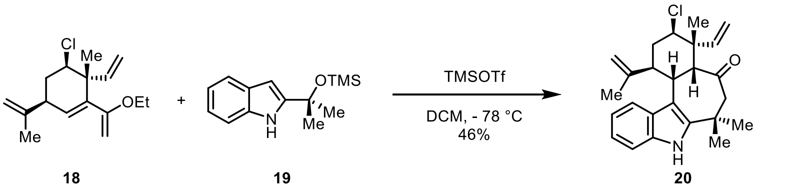

18 19 20

Tetracycle 20. To a flame-dried 50 mL round-bottom flask was added ethoxy diene **18** (534 mg, 2.00 mmol, 2 equiv.), TMS alcohol **19** (248 mg, 1.00 mmol, 1 equiv.) and DCM (10 mL, 0.1 M). The mixture was cooled to - 78 °C, followed by the dropwise addition of TMSOTf (redistilled, 190 µL, 2.10 mmol, 1.05 equiv.). The resulting black solution was stirred at - 78 °C for 30 min. The cooling bath was then removed and H_2O (10 mL) was added in one portion. The biphasic solution was stirred rigorously at room temperature for 15 min before the addition of 1 M HCl aq. (1.5 ml). The resulting biphasic solution was stirred for another 15 min at the same temperature. The two

phases were separated, and the aqueous phase was washed with DCM (3 x 15 mL). The organic phase was combined, washed with $NaHCO_3$ aq (25 mL) and brine (25 mL), dried over Na_2SO_4, filtered and concentrated *in vacuo*. The crude material was purified by flash chromatography twice (1st column, 5% → 15% EtOAc/hexanes, 2nd column, 2% acetone, 30% DCM, 58% hexanes) to afford the desired product tetracycle **20** as a white solid (182 mg, 46%).

R_f= 0.35 (20% EtOAc/hexanes)

^{1}H NMR (500 MHz, $CDCl_3$): δ 7.96 (s, 1H), 7.53 (d, J = 8.0 Hz, 1H), 7.29 (d, J = 8.1 Hz, 1H), 7.15 (ddd, J = 8.0, 7.0, 1.1 Hz, 1H), 7.05 (ddd, J = 8.1, 7.0, 1.0 Hz, 1H), 6.23 (dd, J = 17.7, 11.0 Hz, 1H), 5.44 (dd, J = 11.9, 4.9 Hz, 1H), 5.102-5.01 (m, 2H), 4.77 (app. s, 1H), 4.57-4.52 (m, 1H), 3.76-3.66 (m, 1H), 3.23 (d, J = 4.5 Hz, 1H), 2.80 (d, J = 10.2 Hz, 1H), 2.38 (d, J = 10.2 Hz, 1H), 2.32-2.24 (m, 2H), 2.20-2.14 (m, 1H), 1.55 (s, 3H) , 1.42 (s, 3H) , 1.35 (s, 3H) , 1.13 (s, 3H).

^{13}C NMR (126 MHz, $CDCl_3$): δ 211.4, 146.6, 145.7, 140.5, 133.7, 129.7, 121.9, 119.9, 119.1, 113.5, 112.2, 111.7, 110.3, 65.4, 63.6, 59.1, 45.2, 44.9, 38.7, 36.4, 34.4, 29.9, 29.3, 23.4, 19.6.

IR (thin film): 3439, 3079, 2964, 2926, 1696, 1490, 1344 cm^{-1}

HRMS (ESI) *m/z* calc'd for $C_{25}H_{31}{}^{35}ClNO$ $[M+H]^+$ 369.2094, found 369.2094.

$[\alpha]_D^{19}$= -1.8 ° (c=0.12, $CHCl_3$).

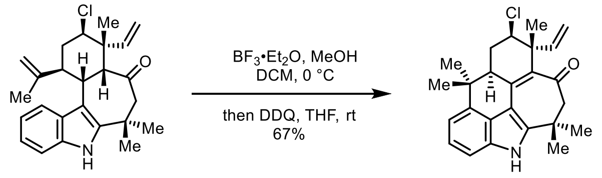

Enone 19. To an oven-dried 25 mL round-bottom flask was added tetracycle **20** (58 mg, 0.146 mmol, 1 equiv.) and DCM (2.43 mL, 0.06 M). The mixture was cooled to 0 °C followed by the addition of $BF_3 \cdot Et_2O$ (72 μL, 0.58 mmol, 4 equiv.), which changed the colorless solution into a dark red solution. MeOH (anhydrous, 6 μL, 0.146 mmol, 1 equiv.) was added and the flask was place into a Cryocooler pre-set at 0 °C. After being stirred at 0 °C for 4 days, TBAF (1 M in THF, 0.659 mL, 0.659 mmol, 4.5 equiv.) was added and the dark red color became less intense. The reaction mixture was stirred at 0 °C for 15 min before H_2O (52 μL, 2.92 mmol, 20 equiv.) was added. The reaction mixture was stirred at the same temperature for another 15 min. DDQ (133 mg, 0.58 mmol, 4 equiv.) was added as a solid and the resulting black solution was removed from the 0 °C cooling bath and stirred at room temperature for 2 hours, before it was quenched with 1 M NaOH aq. (5 mL). The aqueous phase was separated and extracted with DCM (4 x 5 mL). The organic phase was combined, washed with 1 M NaOH (2 x 10 mL), brine (10 mL), dried over $MgSO_4$, filtered and concentrated *in vacuo*. The crude product was purified by flash chromatography (5%→ 15% EtOAc/hexanes) to afford enone **10** as a yellow solid (39 mg, 67%).

R_f= 0.31 (20% EtOAc/hexanes)

^{1}H NMR (500 MHz, $CDCl_3$): δ 8.02(s, 1H), 7.20-7.12 (m, 2H), 7.03 (dd, J = 6.9, 1.2 Hz, 1H), 5.74 (dd, J = 17.5, 10.7 Hz, 1H), 5.21 (dd, J = 10.7, 0.8 Hz, 1H), 5.15 (dd, J = 17.4, 0.8 Hz, 1H), 4.24 (dd, J = 12.9, 3.6 Hz, 1H), 3.21 (d, J = 11.0 Hz, 1H), 2.93 (dd, J = 11.5, 6.7 Hz, 1H), 2.46 (d, J = 11.0 Hz, 1H), 2.40 (ddd, J = 13.1, 6.6, 3.6 Hz, 1H), 2.27 (app. q, J = 12.6 Hz, 1H), 1.65 (s, 3H),

1.54 (s, 3H), 1.49 (s, 3H), 1.33 (s, 3H), 1.04 (s, 3H).

^{13}C NMR (126 MHz, $CDCl_3$): δ 203.9, 144.0, 141.6, 140.1, 138.6, 133.4, 133.0, 125.1, 123.3, 115.6, 113.7, 108.2, 107.1, 67.0, 55.6, 48.3, 45.8, 39.0, 35.9, 30.7, 29.2, 29.0, 25.1, 23.5, 20.1.

HRMS (ESI) *m/z* calc'd for $C_{25}H_{39}{}^{35}ClNO$ $[M+H]^+$ 376.1832, found 376.1825.

IR (thin film): 3364, 2966, 1670, 1448, 1466, 1367, 1320, 1176, 756 cm^{-1}

$[\alpha]_D^{19}$= +51.3 ° (c=0.11, $CHCl_3$).

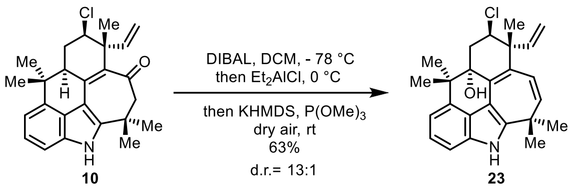

Pentacyclic alcohol 23. To a flame-dried 25 mL round-bottom flask was added enone **10** (8 mg, 0.0203 mmol, 1 equiv.) and DCM (2.03 mL, 0.01 M). The pale-yellow solution was cooled to - 78 °C before the dropwise addition of DIBAL (1 M in PhMe, 81 μL, 0.0812 mmol, 4 equiv.). The resulting orange solution was stirred at - 78 °C for 1.5 h before the dropwise addition of HCl (4M in dioxane, 18 μL, 0.0711 mmol, 3.5 equiv.), followed by Et_2AlCl (1 M in hexanes, 20 μL, 0.0203 mmol, 1 equiv.). After 15 min at - 78 °C, the dry-ice acetone bath was changed to ice water bath and the reaction was stirred at 0 °C for 10 min, during which time the reaction turned red. $P(OMe)_3$ (17 μL, 0.143 mmol, 7 equiv.) was added and the red color faded into yellow. The reaction mixture was then cooled to – 78 °C. KHMDS (1 M in THF, 102 μL, 0.102 mmol, 5 equiv.) was added and the cooling bath was removed. Dry air was bubbled through the reaction mixture as it warmed to room temperature. The bubbling continued for a total of 20 min while the reaction was at room temperature before 1 M NaOH (50 μL) was added. The reaction mixture was stirred at room temperature for another15 min before the addition of $MgSO_4$. The resulting suspension was stirred for an additional 15 min before filtration. The filtrate was concentrated *in vacuo* and purified by flash chromatography (2%→ 8% EtOAc/hexanes) to afford a mixture of **23** and its diastereomer as a white solid (5 mg, 63%). The resulting solid was further purified by preparative TLC (4% acetone, 30% DCM, 56% hexanes) to afford **23** (3.7 mg) as a white solid.

R_f= 0.33 (20% EtOAc/hexanes)

^{1}H NMR (500 MHz, $CDCl_3$): δ 7.96 (s, 1H), 7.23-7.19 (m, 2H), 7.09 (dd, *J* = 4.9, 3.0 Hz, 1H), 5.92 (d, *J* = 11.5 Hz, 1H), 5.76 (dd, *J* = 17.4, 10.7 Hz, 1H), 5.41 (d, *J* = 11.5 Hz, 1H), 5.29 (dd, *J* = 10.7, 1.0 Hz, 1H), 5.23 (dd, *J* = 17.4, 1.0 Hz, 1H), 4.51 (dd, *J* = 12.9, 3.8 Hz, 1H), 2.59 (td, *J* = 13.3, 2.1 Hz, 1H), 2.46 (dd, *J* = 13.6, 3.8 Hz, 1H), 2.04 (app. d, *J* = 2.3 Hz, 1H) , 1.67 (s, 3H) , 1.60 (s, 3H) , 1.46 (s, 3H) , 1.11 (s, 3H) , 1.02 (s, 3H).

^{13}C NMR (126 MHz, $CDCl_3$): δ 144.4, 140.9, 137.0, 133.9, 133.0, 132.4, 130.2, 127.7, 123.8, 123.7, 115.7, 115.4, 108.9, 106.8, 78.0, 63.8, 46.8, 45.3, 36.4, 35.6, 28.2, 26.6, 26.2, 18.2, 17.5.

HRMS (ESI) *m/z* calc'd for $C_{25}H_{27}{}^{35}ClNO$ $[M+H-H_2O]^+$ 376.1832, found 376.1825.

IR (thin film): 3536, 3344, 2969, 1570, 1473, 1458, 1458, 1361, 1308, 1266, 911, 744, 707 cm^{-1}

$[\alpha]_D^{19}$= +8.7 ° (c=0.17, $CHCl_3$).

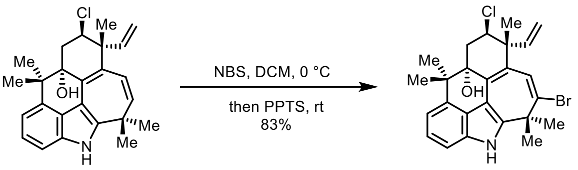

23 **24**

Bromide 24. To an oven dried 4 mL vial was added **23** (2.0 mg, 0.0051 mmol, 1 equiv.) and DCM (250 μL). The colorless solution was cooled to 0 °C. *N*-bromosuccinimide (0.9 mg, 0.0051 mmol, 1 equiv.) was added as a DCM solution (50 μL of a stock solution made by dissolving 9 mg of *N*-bromosuccinimide in 500 μL DCM). The resulting orange solution was stirred at 0 °C for 10 min before the addition of pyridinium *p*-toluenesulfonate (2.6 mg, 0.010 mmol, 2 equiv.) as a DCM solution (100 μL of a stock solution made by dissolving 13 mg pyridinium *p*-toluenesulfonate in 500 μL DCM). The red solution was stirred at 0 °C and then at room temperature. The reaction was stirred at this temperature for 90 minutes before being quenched by $NaHCO_3$ aq. (500 μL). The two phases were separated, and the aqueous phase was extracted with DCM (3 x 1 mL). The organic phase was combined, dried over Na_2SO_4, filtered and concentrated *in vacuo*. The crude product was purified by preparative TLC (40% EtOAc/hexanes) to afford bromide **24** as a yellow solid (1.8 mg, 83%).

R_f= 0.30 (20% EtOAc/hexanes)
^{1}H NMR (500 MHz, $CDCl_3$): δ 8.03 (s, 1H), 7.26-7.22 (m, 2H), 7.11 (dd, *J* = 5.6, 2.2 Hz, 1H), 6.61 (s, 3H), 5.73 (dd, *J* = 17.4, 10.6 Hz, 1H), 5.33 (d, *J* = 10.6 Hz, 1H), 5.26 (d, *J* = 17.4 Hz, 1H), 4.47 (dd, *J* = 12.9, 3.7 Hz, 1H), 2.58 (app. t, *J* = 13.4 Hz, 1H), 2.45 (dd, *J* = 13.7, 3.8 Hz, 1H), 2.01 (s, 1H) , 1.90 (s, 3H) , 1.60 (s, 3H) , 1.47 (s, 3H) , 1.11 (s, 3H) , 1.09 (s, 3H).
^{13}C NMR (126 MHz, $CDCl_3$): δ 143.6, 137.3, 137.1, 133.5, 132.9, 131.5, 130.5, 124.5, 123.5, 123.2, 116.1, 115.9, 109.2, 108.0, 77.9, 63.3, 46.8, 45.5, 41.4, 36.3, 28.7, 28.3, 25.0, 18.1, 17.4.
HRMS (ESI) *m/z* calc'd for $C_{25}H_{26}{}^{79}Br^{35}ClN$ $[M+H-H_2O]^+$ 4543.0937, found 454.0929.
IR (thin film): 3522, 3334, 2966, 1559, 1472, 1457, 1362, 1308, 923, 760 cm^{-1}
$[\alpha]_D^{26}$= +23.0 ° (c=0.15, $CHCl_3$).

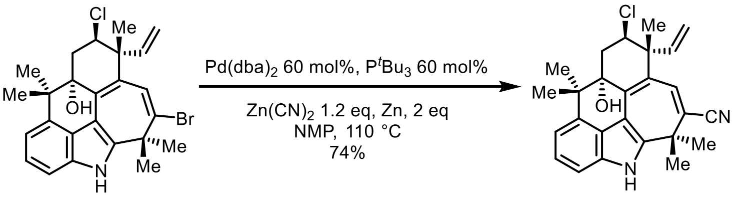

24 **25**

Nitrile 25. Tri-*tert*-butyl phosphine (12.3 mg) was weighed into an oven-dried 4 mL vial in a glove box. The vial was then capped and taken out of the glove box. *N*-Methylpyrrolidinone (NMP, bubbled with N_2 for 40 minutes before use, 958 μl) was added into the vial to make a colorless solution. To different oven-dried 4 mL vial was added $Pd(dba)_2$ (11.0 mg) and NMP (bubbled with N_2 for 40 minutes before use, 300 μL). The dark red solution was purged with N_2 for 3 min, before 300 μL of the tri-*tert*-butyl phosphine NMP solution was added. Another 150 μL of NMP was added and the mixture was stirred at room temperature for 1 hour.

To an oven-dried 4 mL vial was added bromide **24** (3.0 mg, 0.0063 mmol, 1 equiv.), $Zn(CN)_2$

(0.9 mg, 0.0076 mmol, 1.2 equiv.), Zn dust (3.3 mg, 0.0508 mmol, 8 equiv. *Note 1*) and NMP (bubbled with N_2 for 40 minutes before use, 270 μL). The vial was purged with N_2 for 3 min. The mixture was heated to 110 °C and the pre-prepared $Pd(dba)_2$-P^tBu_3 NMP solution (50 μL, contained $Pd(dba)_2$ 0.73 mg, 0.0013 mmol, 0.2 equiv.; P^tBu_3 0.26 mg, 0.0013 mmol, 0.2 equiv.) was added into the vial containing **24** with rigorous stirring. The resulting clear brown solution was stirred at this temperature for 40 minutes. Another 100 μL of the $Pd(dba)_2$-P^tBu_3 NMP solution (contained $Pd(dba)_2$ 1.46 mg, 0.0026 mmol, 0.4 equiv.; P^tBu_3 0.51 mg, 0.0026 mmol, 0.4 equiv.) was added and the brown solution was stirred at the same temperature for an extra 40 minute. The reaction mixture was then filter through a pad of celite and flashed with EtOAc. The organic phase was washed with H_2O (3 x 1 mL), brine (1 mL), dried over Na_2SO_4, filtered and concentrated *in vacuo*. The crude material was purified by prep-TLC (50% EtOAc/hexanes) to afford the desired product nitrile **25** as a yellow solid (2.0 mg, 74%).

Note 1: The ratio of Zn dust to $Pd(dba)_2$ needs to be at least 10 to 1 to ensure reproducible results.

R_f= 0.14 (20% EtOAc/hexanes)
^{1}H NMR (500 MHz, $CDCl_3$): δ 8.17 (s, 3H), 7.30-7.25 (m, 2H7.14 (dd, *J* = 5.4, 2.5 Hz, 1H), 6.78 (s, 3H), 5.72 (dd, *J* = 17.4, 10.6 Hz, 1H), 5.35 (d, *J* = 10.6 Hz, 1H), 5.28 (d, *J* = 17.4 Hz, 1H), 4.46 (dd, *J* = 12.9, 3.7 Hz, 1H), 2.60 (t, J = 13.3 Hz, 1H), 2.47 (dd, *J* = 13.7, 3.8 Hz, 1H), 2.07 (s, 3H), 1.93 (s, 3H) , 1.61 (s, 3H) , 1.50 (s, 3H) , 1.11 (s, 3H) , 1.08 (s, 3H).
^{13}C NMR (125 MHz, $CDCl_3$): δ 143.3, 142.2, 139.7, 136.7, 136.3, 133.1, 132.7, 125.0, 123.1, 119.5, 116.7, 116.6, 111.0, 109.5, 107.6, 78.0, 62.7, 46.7, 45.5, 36.2, 35.7, 28.0, 25.1, 25.0, 18.0, 17.7.
HRMS (ESI) *m/z* calc'd for $C_{26}H_{26}{}^{35}ClN_2$ $[M+H-H_2O]^+$ 401.1785, found 401.1777.
IR (thin film): 3333, 2972, 2927, 2206, 1559, 1535, 1471, 1363, 1309, 1265, 1078, 1054, 914, 761, 736 cm^{-1}
$[\alpha]_D^{19}$= +82.2 ° (c=0.13, $CHCl_3$).

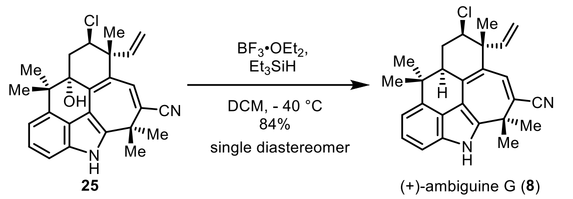

25

(+)-ambiguine G (**8**)

(+)-Ambiguine G (8). To a flame-dried 10 mL round-bottom flask containing nitrile **25** (2.6 mg, 0.0062 mmol, 1 equiv.) was added DCM (478 μL, 0.013 M), followed by Et_3SiH (20 μL, 0.124 mmol, 20 equiv.). The resulting yellow solution was cooled to - 40 °C. $BF_3{\cdot}OEt_2$ (3.8 μL, 0.0310 mmol, 5 equiv.) was added to the reaction mixture slowly as a DCM solution (50 μL, made by dissolving 38 μL $BF_3{\cdot}OEt_2$ in 462 μL DCM), which caused the reaction to turn into a light brown, opaque solution. The reaction was stirred at – 40 °C for 30 min and quenched with saturated $NaHCO_3$ aq. (2 mL) at the same temperature. The resulting mixture was then allowed to warm to rt and extracted with DCM (2 x 2 mL). The organic phase was washed with saturated $NaHCO_3$ aq. (1 x 2 mL), dried with Na_2SO_4, filtered and concentrated *in vacuo*. The crude product was purified by prep-TLC (25% EtOAc/hexanes) to afford (+)-ambiguine G (**8**) as a yellow solid (2.1 mg, single diastereomer, 84%).

R_f= 0.29 (20% EtOAc/hexanes)

^{1}H NMR (500 MHz, CD_3OD): δ 8.04 (s, 1H), 7.25-7.20 (m, 2H), 7.08 (dd, *J* = 5.5, 2.3 Hz, 1H), 6.76 (s, 3H), 5.70 (dd, *J* = 17.4, 10.6 Hz, 1H), 5.32 (d, *J* = 10.6 Hz, 1H), 5.26 (d, *J* = 17.3 Hz, 1H), 4.19 (dd, *J* = 12.9, 3.8 Hz, 1H), 3.18 (dd, *J* = 11.4, 7.4 Hz, 1H), 2.44 (ddd, *J* = 13.4, 7.4, 3.8 Hz, 1H), 2.34 (app. q, *J* = 12.6 Hz, 1H) , 1.91 (s, 3H) , 1.56 (s, 3H) , 1.52 (s, 3H) , 1.06 (s, 3H) , 1.04 (s, 3H).

^{13}C NMR (125 MHz, CD_3OD): δ 143.9, 142.3, 139.7, 137.5, 135.9, 133.0, 132.5, 124.7, 124.2, 119.9, 116.3, 114.6, 110.3, 109.5, 109.0, 65.5, 48.1, 46.3, 40.3, 35.5, 30.3, 25.1, 24.8, 24.8, 23.4, 18.8.

HRMS (ESI) *m/z* calc'd for $C_{26}H_{28}{}^{35}ClN_2$ $[M+H]^+$ 403.1941, found 403.1930.

IR (thin film): 3343, 2974, 2202, 1653, 1539, 1472, 1457, 1362, 1315 cm^{-1}

$[\alpha]_D^{20}$= +143.2 ° (c=0.05, $CHCl_3$).

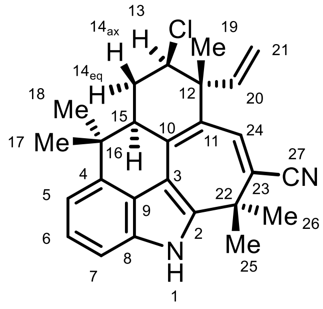

(+)-Ambiguine G

Table S1: Comparison of the ^{1}H NMR data of synthetic ambiguine G with reported data

Position	Synthetic ambiguine G, in $CDCl_3$, δ_H, mult. (J in Hz)	Reported[2] chemical shift values, in $CDCl_3$, δ_H, mult. (J in Hz)
N-H	8.04, s	8.06, s
5	7.08, dd (5.5, 2.3)	7.07, dd (8.2, 0.6)
6	7.25-7.20, m, overlapped with H-7	7.24, dd (8.2, 7.5)
7	7.25-7.20, m, overlapped with H-6	7.22, dd (7.5, 0.6)
13	4.19, dd (12.9, 3.8)	4.19, dd (12.7, 3.9)
14_{ax}	2.34, app. q (12.6)	2.34, q (13.3, 12.7, 11.2)
14_{eq}	2.44, ddd (13.4, 7.4, 3.8)	2.44, ddd (13.3, 7.5, 3.9)
15	3.18, dd (11.4, 7.4)	3.18, dd (11.2, 7.5)
17	1.06, s	1.06, s
18	1.56, s	1.56, s
19	1.52, s	1.52, s
20	5.70, dd (17.4, 10.6)	5.71, dd (17.4, 10.8)
21E	5.32, d (10.6)	5.32, d (10.8)
21Z	5.26, d (17.3)	5.26, d (17.4)
24	6.76, s	6.76, s
25	1.91, s	1.91, s
26	1.04, s	1.04, s

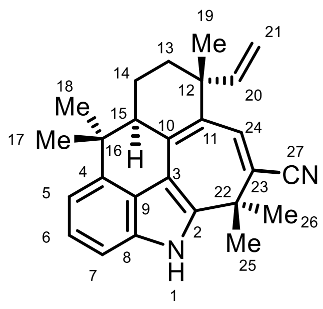

(+)-Ambiguine G

Table S2: Comparison of the ^{13}C NMR data of synthetic ambiguine G with reported data

Position	Synthetic ambiguine G (**CS1**), in $CDCl_3$, δ_C (ppm)	Reported[2] chemical shift values (**CS2**), in $CDCl_3$, δ_C (ppm)	Δδ (**CS1**-**CS2**, ppm)
2	137.5	137.4	0.1
3	110.3	110.1	0.2
4	139.7	139.5	0.2
5	114.6	114.4	0.2
6	124.2	124.1	0.1
7	109.0	108.8	0.2
8	133.0	132.9	0.1
9	124.7	124.5	0.2
10	135.9	135.7	0.2
11	132.5	132.3	0.2
12	46.3	46.2	0.1
13	65.5	65.3	0.2
14	30.3	30.1	0.2
15	48.1	47.9	0.2
16	40.3	40.1	0.2
17	24.8	24.7	0.1
18	23.4	23.2	0.2
19	18.8	18.6	0.2
20	143.9	143.8	0.1
21	116.3	116.1	0.2
22	35.5	35.3	0.2
23	109.5	109.3	0.2
24	142.3	142.1	0.2
25	24.8	24.6	0.2
26	25.1	24.9	0.2
27	119.9	119.7	0.2

III. References

(1) Xu, J.; Rawal, V. H. Total synthesis of (-)-ambiguine P. *J. Am. Chem. Soc*. **2019**, *141*, 4820-4823.

(2) Huber, U.; Moore, R. E.; Patterson, G. M. L. Isolation of a nitrile-containing indole alkaloid from the terrestrial blue-green alga *Hapalosiphon delicatulus*. *J. Nat. Prod.* **1998**, *61*, 1304-1306

IV. NMR Spectra

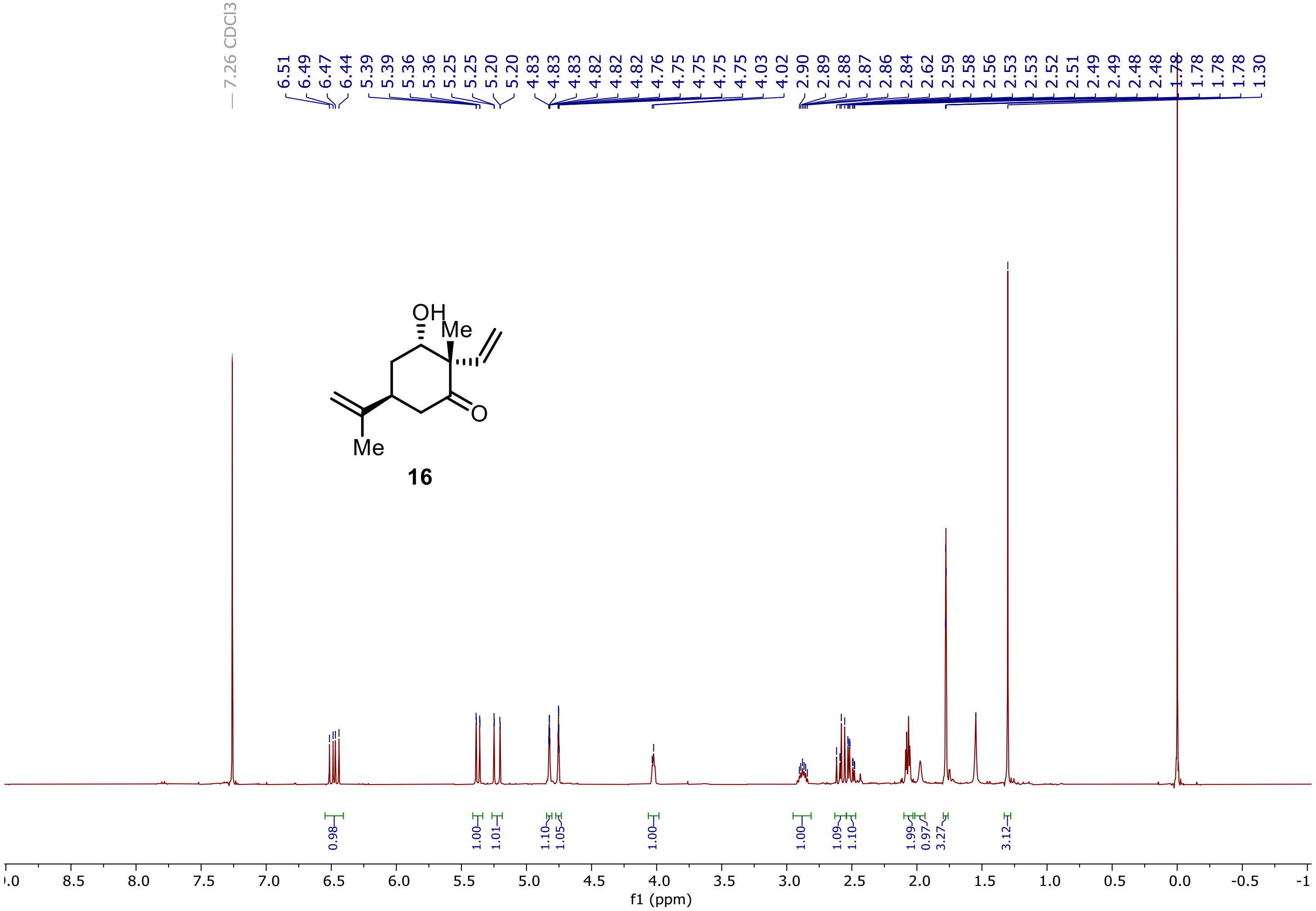

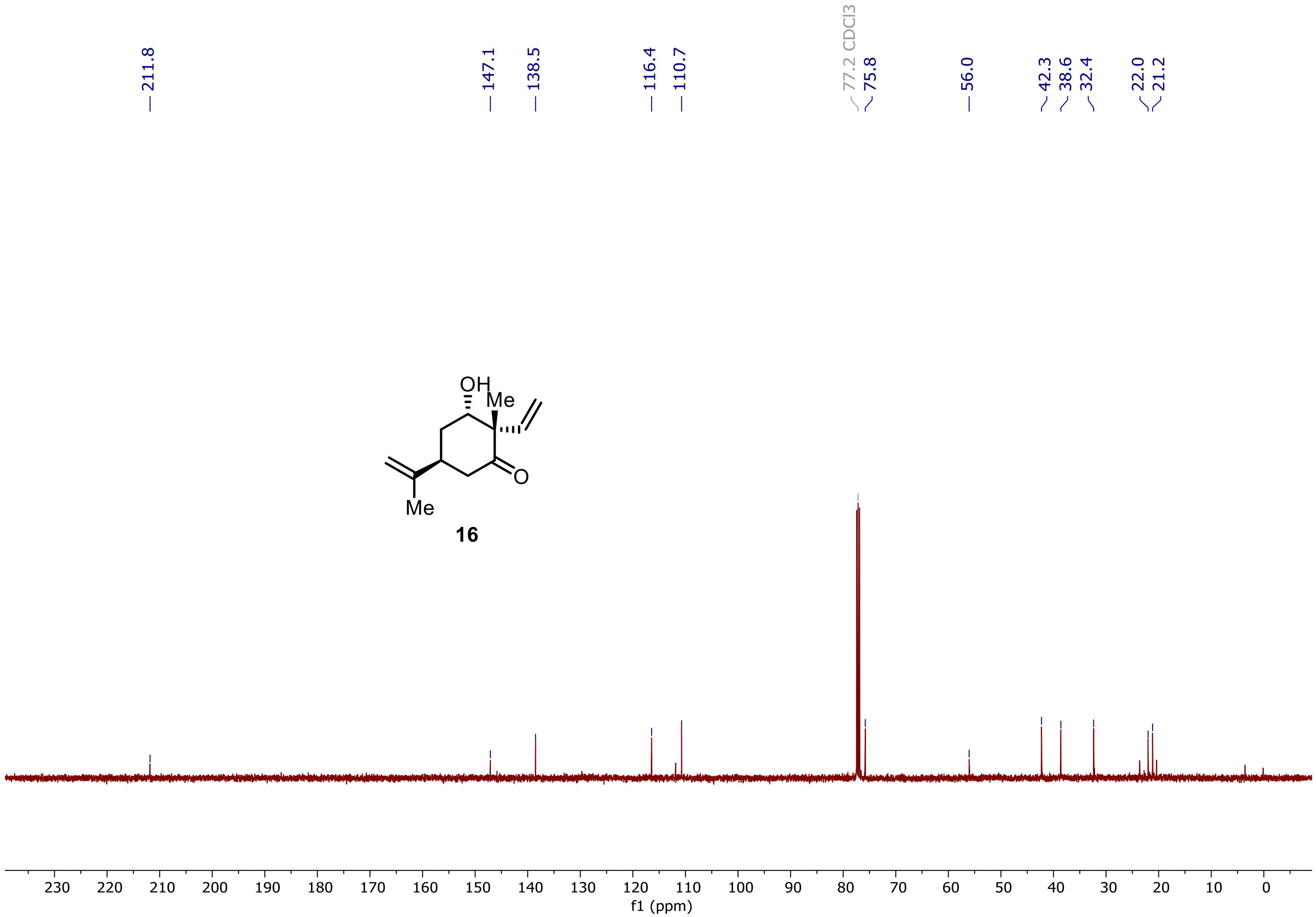
211.8
147.1
138.5
116.4
110.7
77.2 CDCl3
75.8
56.0
42.3
38.6
32.4
22.0
21.2
OH
Me
O
Me
16
230 220 210 200 190 180 170 160 150 140 130 120 110 100 90 80 70 60 50 40 30 20 10 0
f1 (ppm)

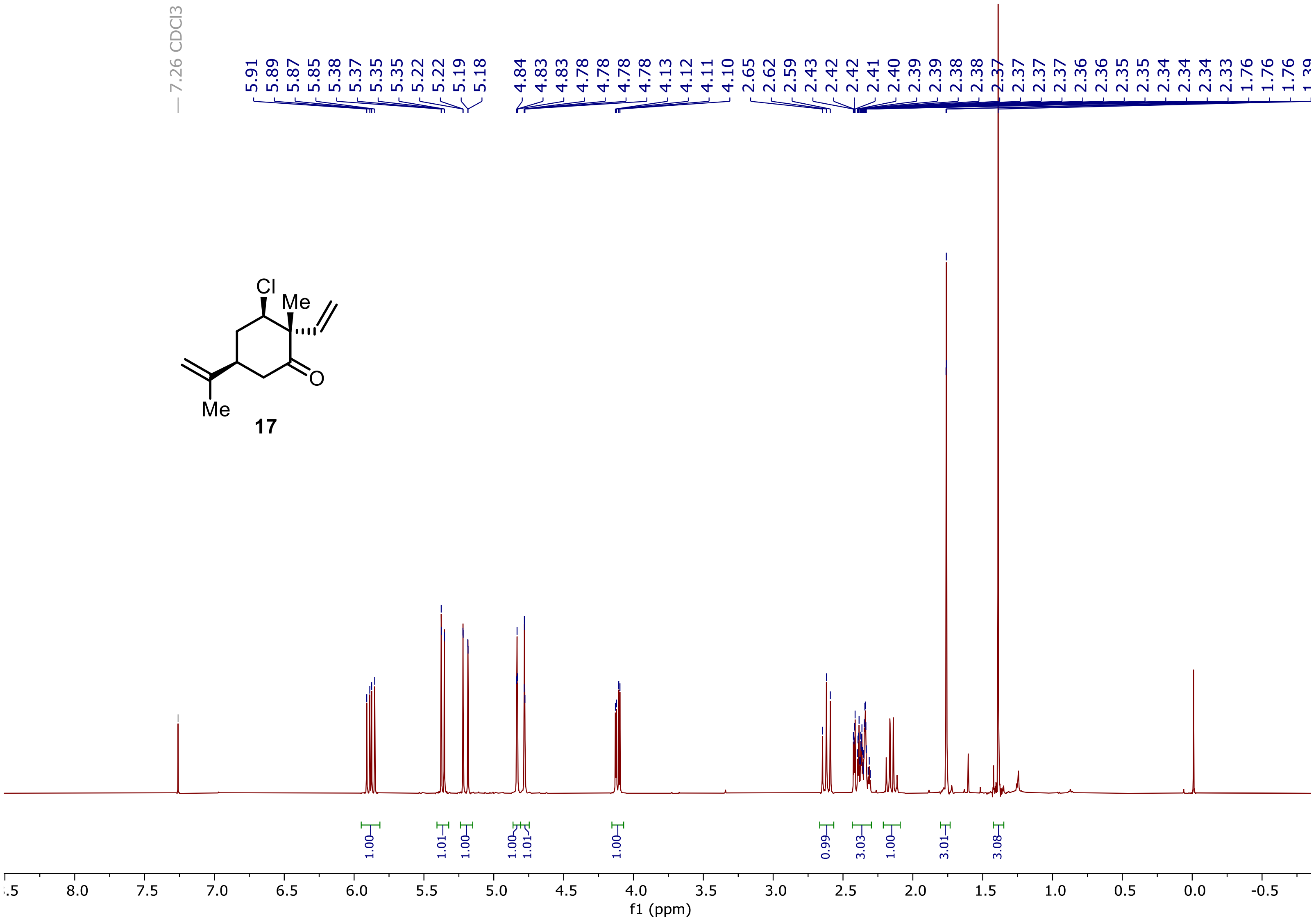

17

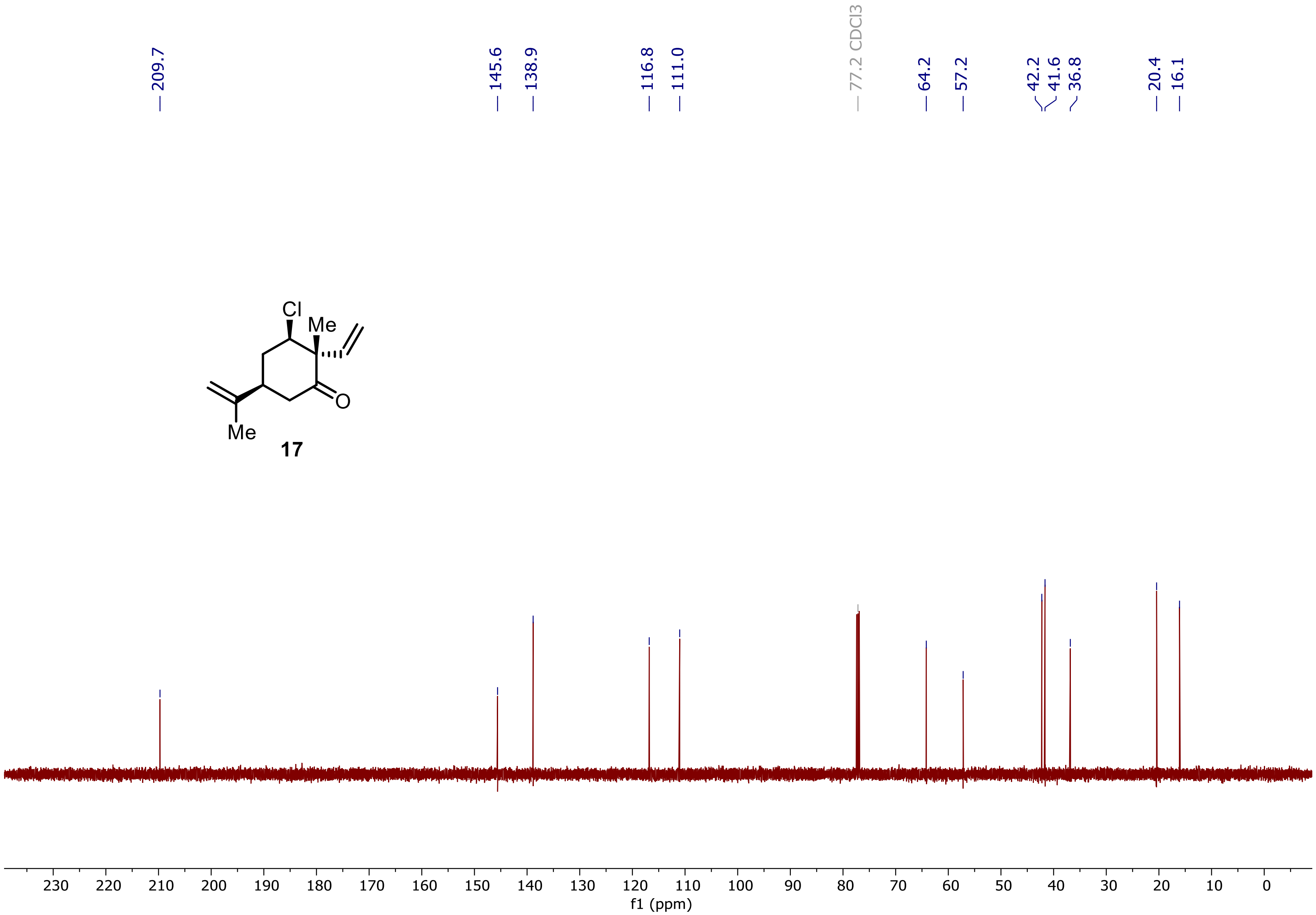

209.7
145.6
138.9
116.8
111.0
77.2 CDCl3
64.2
57.2
42.2
41.6
36.8
20.4
16.1
Cl
Me
O
Me
17
230 220 210 200 190 180 170 160 150 140 130 120 110 100 90 80 70 60 50 40 30 20 10 0
f1 (ppm)

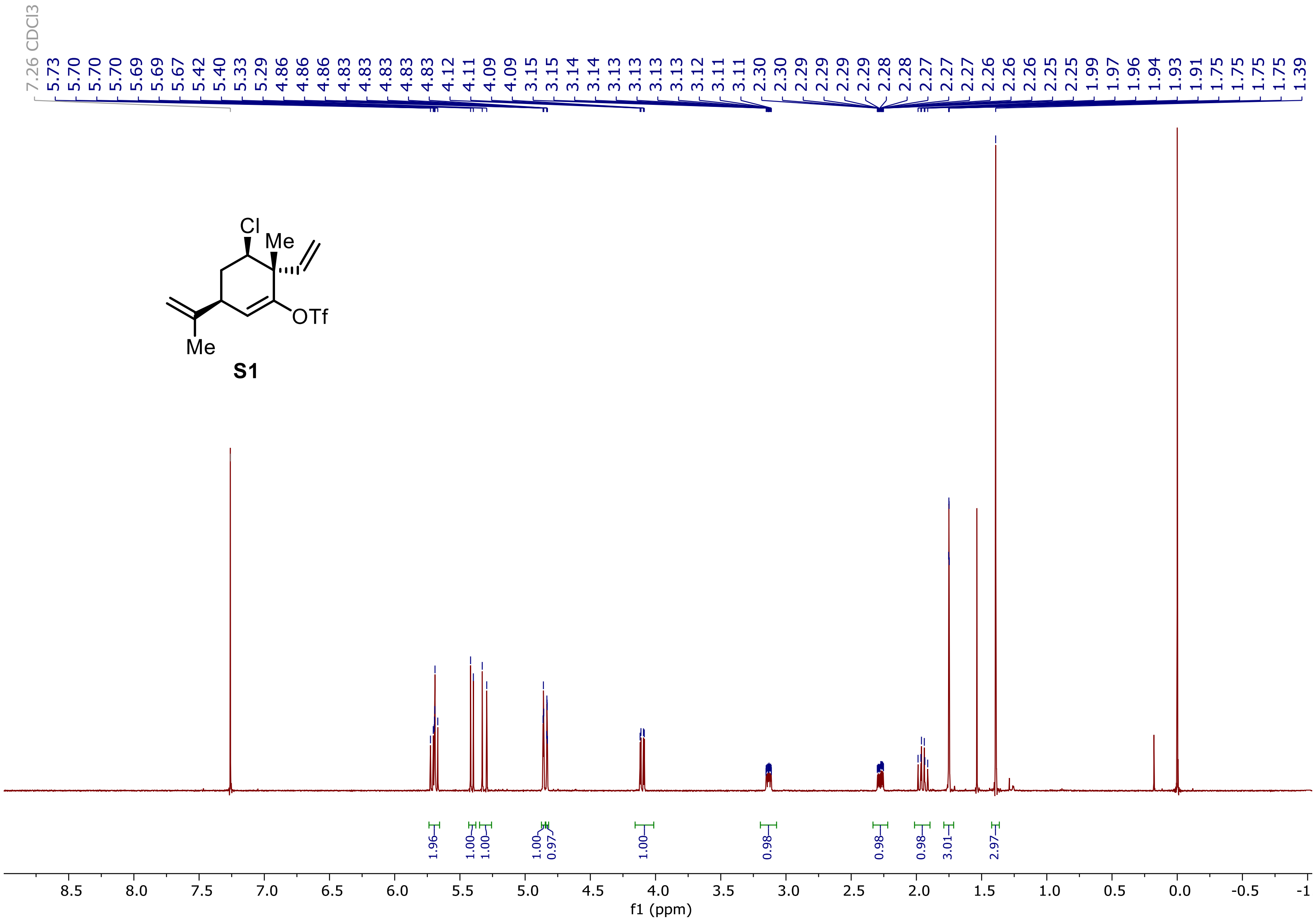
7.26 CDCl3
5.73 5.70 5.70 5.70 5.69 5.69 5.67 5.42 5.40 5.33 5.29 4.86 4.86 4.86 4.83 4.83 4.83 4.83 4.83 4.12 4.11 4.09 4.09 3.15 3.15 3.14 3.14 3.13 3.13 3.13 3.13 3.12 3.11 3.11 2.30 2.30 2.29 2.29 2.29 2.29 2.28 2.28 2.27 2.27 2.27 2.26 2.26 2.26 2.25 2.25 1.99 1.97 1.96 1.94 1.93 1.91 1.75 1.75 1.75 1.75 1.39
Cl
Me
OTf
Me
S1
1.96
1.00
1.00
1.00
0.97
1.00
0.98
0.98
0.98
3.01
2.97
8.5 8.0 7.5 7.0 6.5 6.0 5.5 5.0 4.5 4.0 3.5 3.0 2.5 2.0 1.5 1.0 0.5 0.0 -0.5 -1
f1 (ppm)

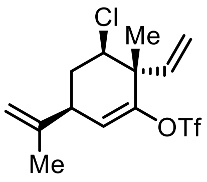
Cl
Me
OTf
Me

S1

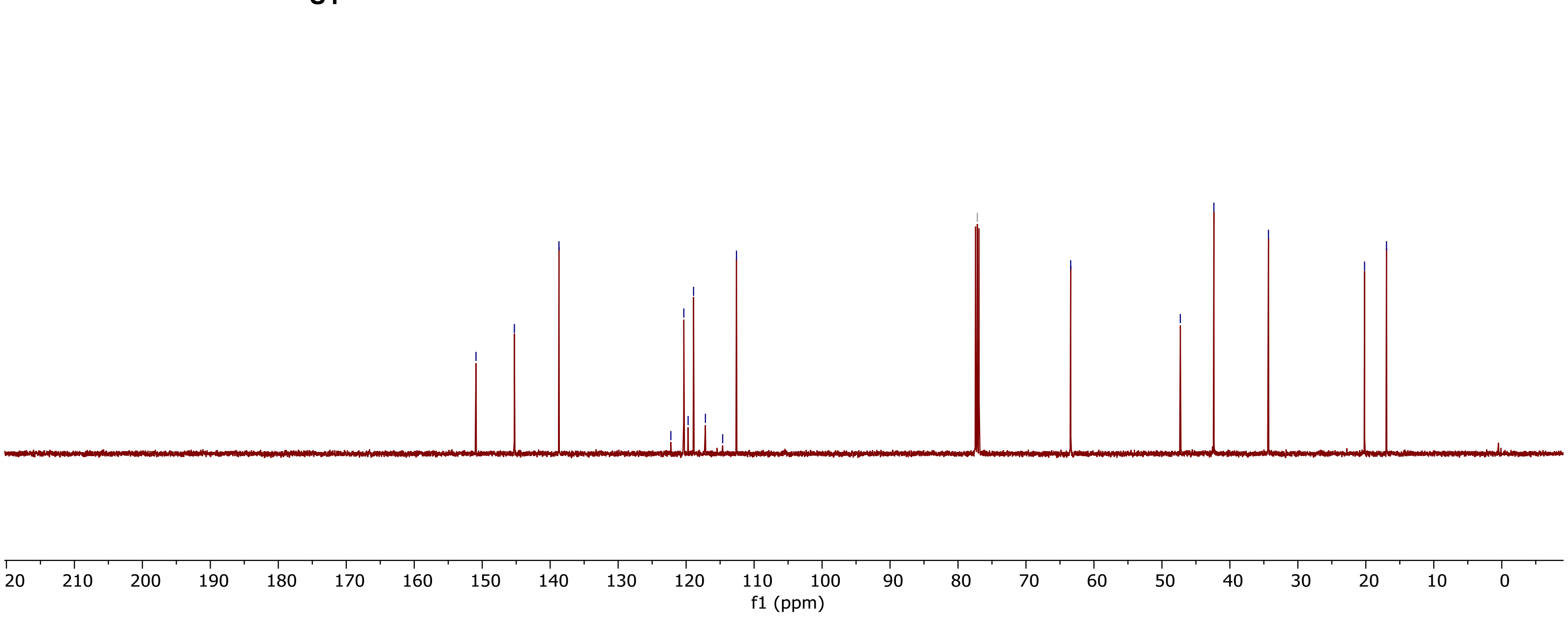
150.9
145.3
138.7
122.2
120.3
119.7
118.9
117.2
114.6
112.6
77.2 CDCl3
63.4
47.3
42.4
34.3
20.2
17.0
20
210
200
190
180
170
160
150
140
130
120
110
100
90
80
70
60
50
40
30
20
10
0
f1 (ppm)

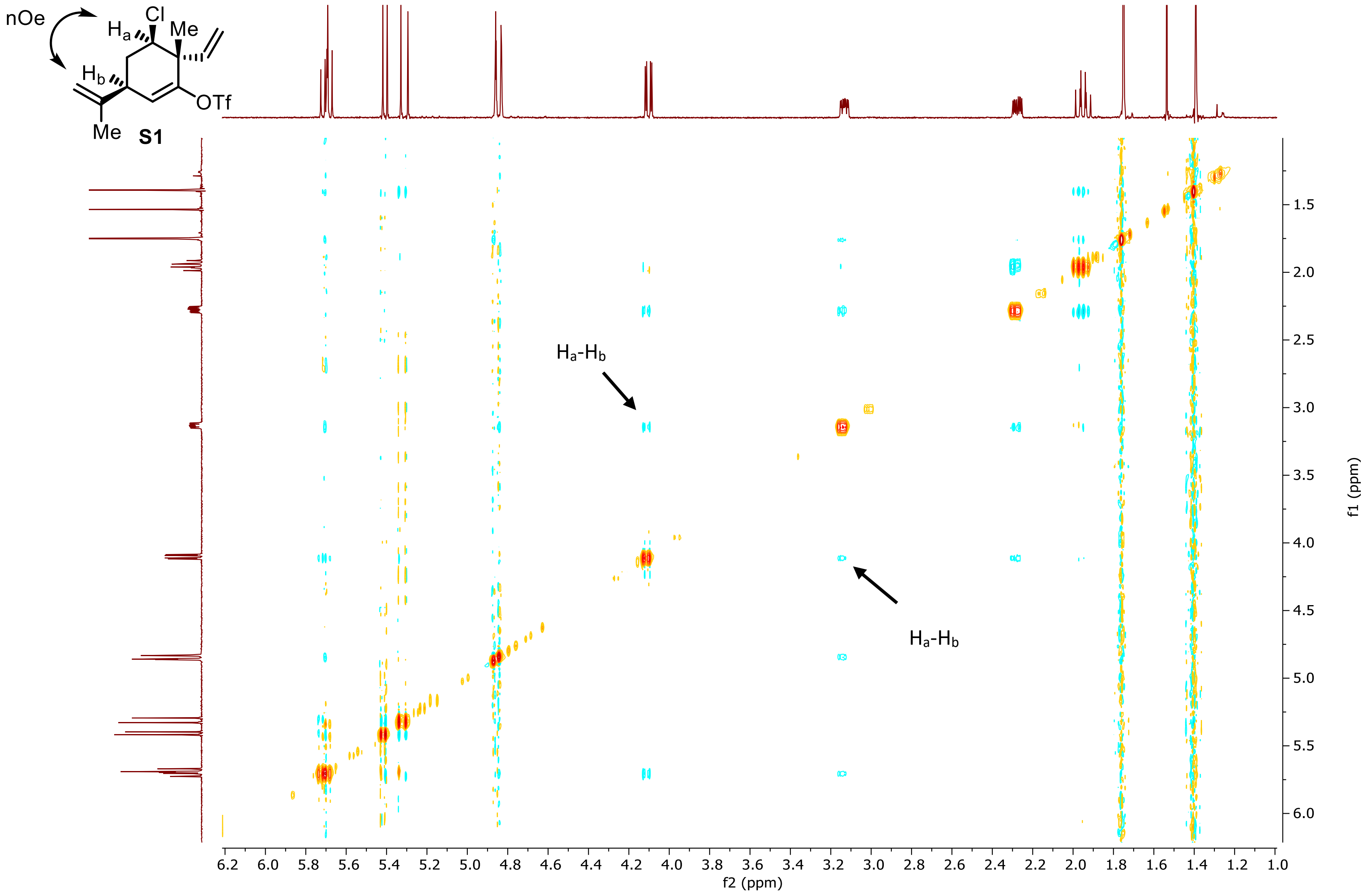
nOe
Cl
Ha
Me
Hb
OTf
Me
S1
Ha-Hb
Ha-Hb
f2 (ppm)
f1 (ppm)

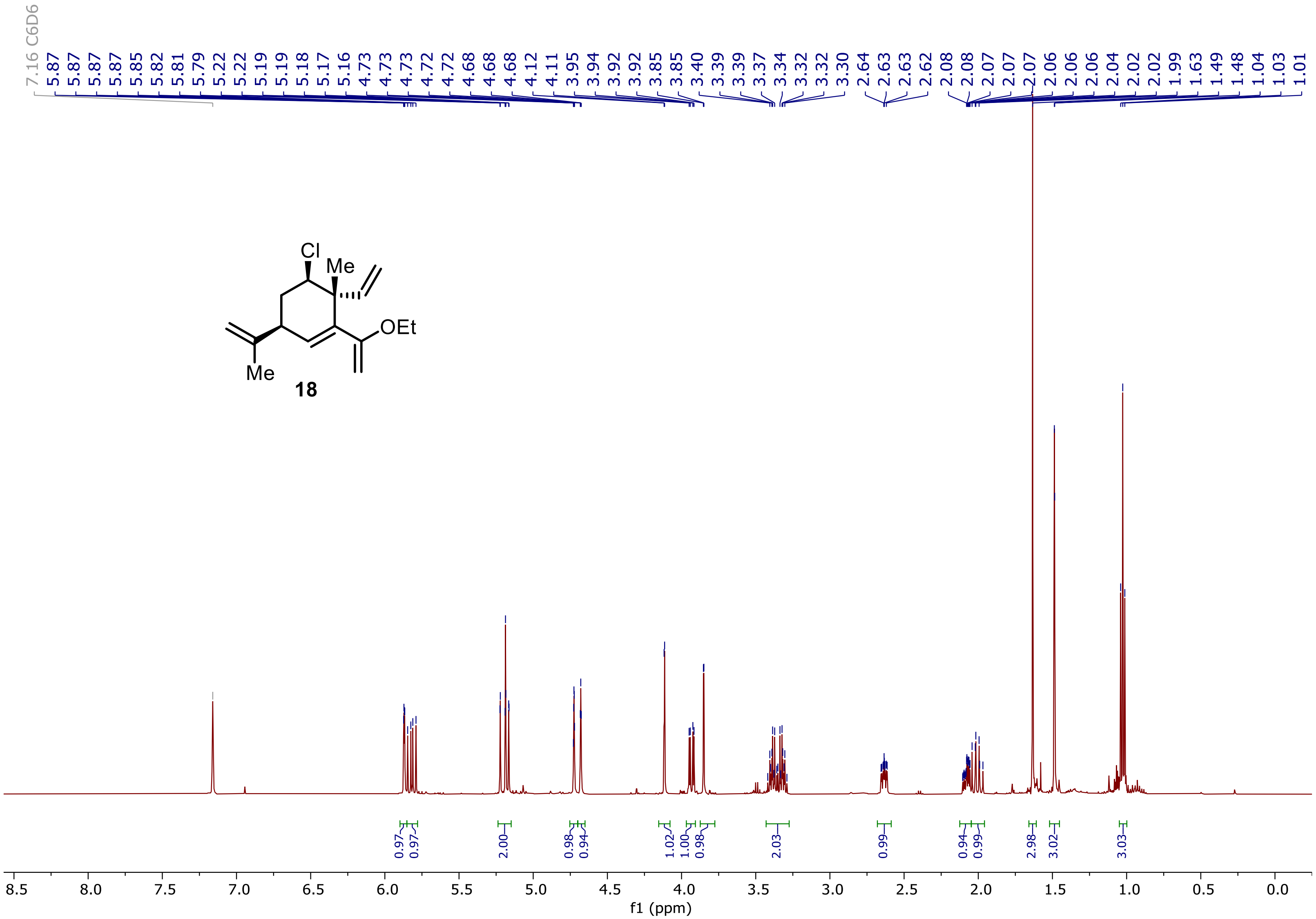
7.16 C6D6
5.87
5.87
5.87
5.87
5.85
5.82
5.81
5.79
5.22
5.22
5.19
5.19
5.18
5.17
5.16
4.73
4.73
4.72
4.72
4.68
4.68
4.68
4.12
4.11
3.95
3.94
3.92
3.92
3.85
3.85
3.40
3.39
3.39
3.37
3.34
3.32
3.32
3.30
2.64
2.63
2.63
2.62
2.08
2.08
2.07
2.07
2.07
2.06
2.06
2.06
2.04
2.02
2.02
1.99
1.63
1.49
1.48
1.04
1.03
1.01
Cl
Me
OEt
Me
18
0.97
0.97
2.00
0.98
0.94
1.02
1.00
0.98
2.03
0.99
0.94
0.99
2.98
3.02
3.03
8.5
8.0
7.5
7.0
6.5
6.0
5.5
5.0
4.5
4.0
3.5
3.0
2.5
2.0
1.5
1.0
0.5
0.0
f1 (ppm)

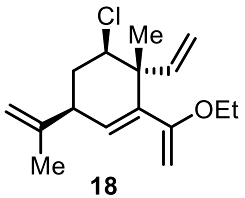

18

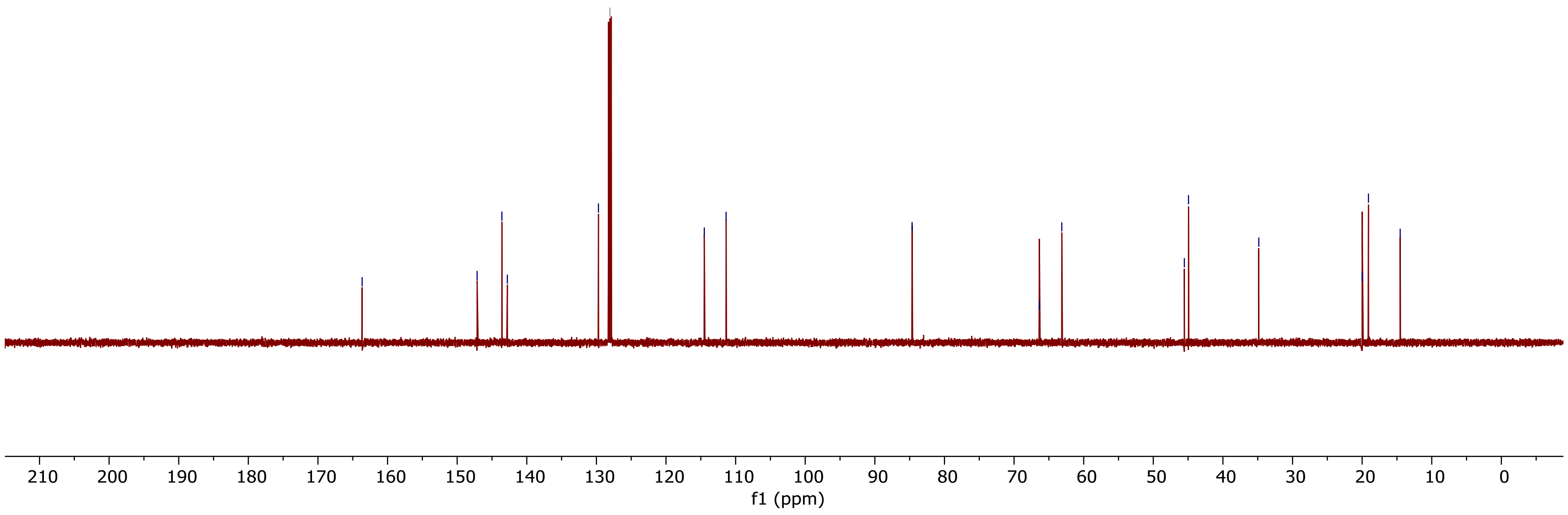

163.7
147.1
143.6
142.8
129.7
128.1 C6D6
114.5
111.4
84.6
66.4
63.1
45.5
44.9
34.8
20.0
19.1
14.5
210 200 190 180 170 160 150 140 130 120 110 100 90 80 70 60 50 40 30 20 10 0
f1 (ppm)

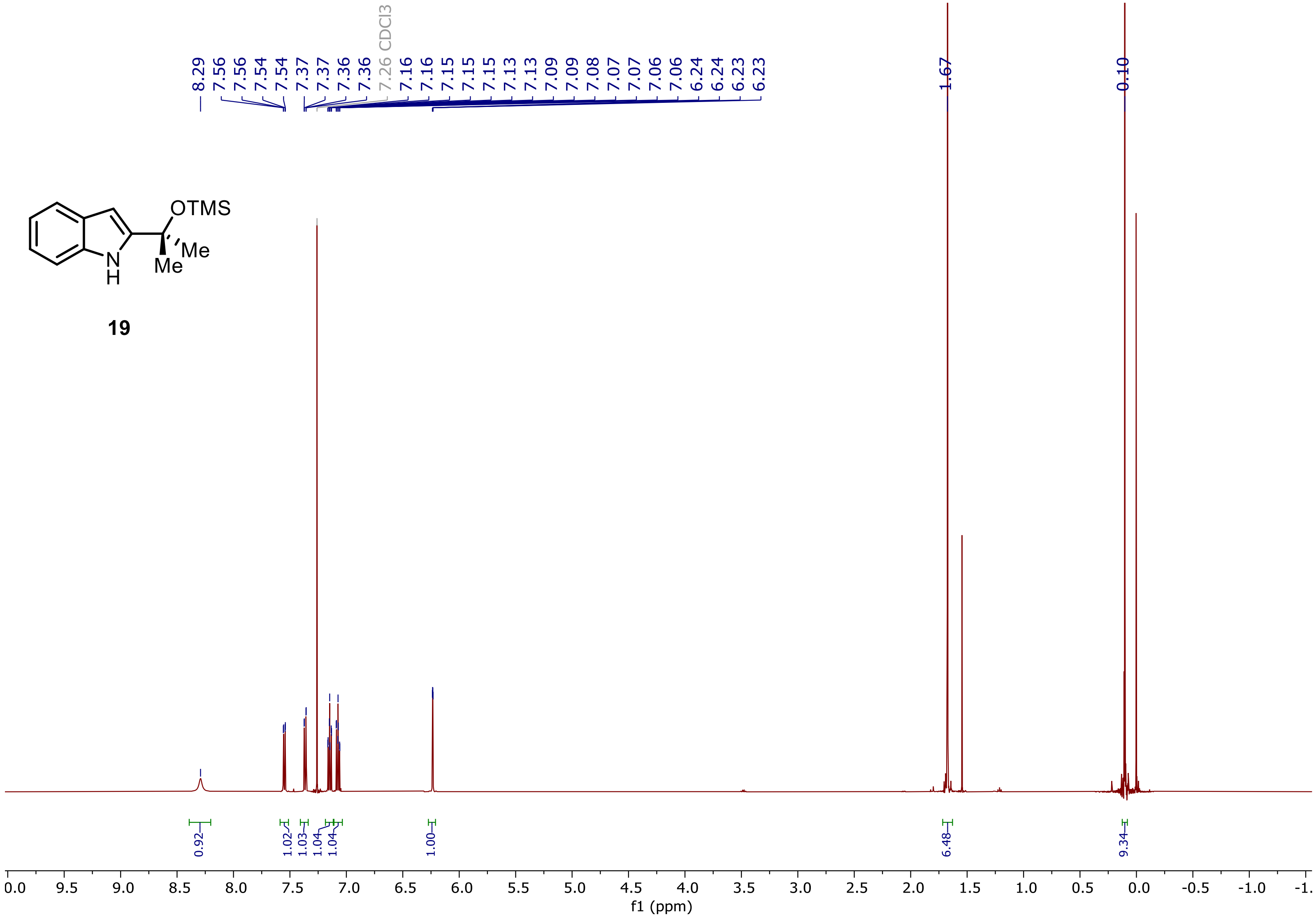

OTMS
Me
Me
N
H
19
8.29
7.56
7.56
7.54
7.54
7.37
7.37
7.36
7.36
7.26 CDCl3
7.16
7.16
7.15
7.15
7.15
7.13
7.13
7.09
7.09
7.08
7.07
7.07
7.06
7.06
6.24
6.24
6.23
6.23
1.67
0.10
0.92
1.02
1.03
1.04
1.04
1.00
6.48
9.34
f1 (ppm)

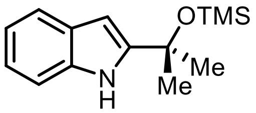

19

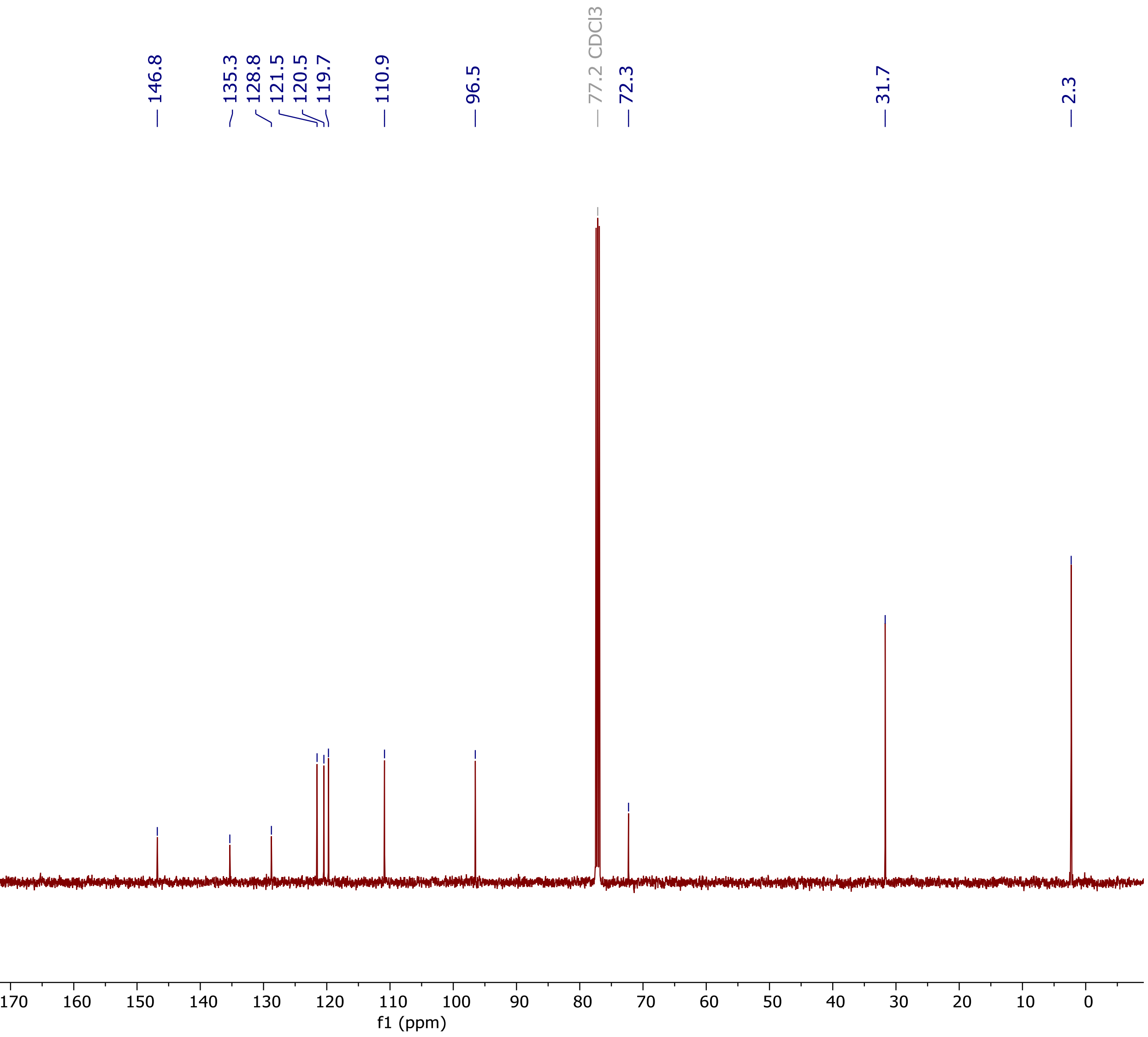
146.8
135.3
128.8
121.5
120.5
119.7
110.9
96.5
77.2 CDCl3
72.3
31.7
2.3
220 210 200 190 180 170 160 150 140 130 120 110 100 90 80 70 60 50 40 30 20 10 0
f1 (ppm)

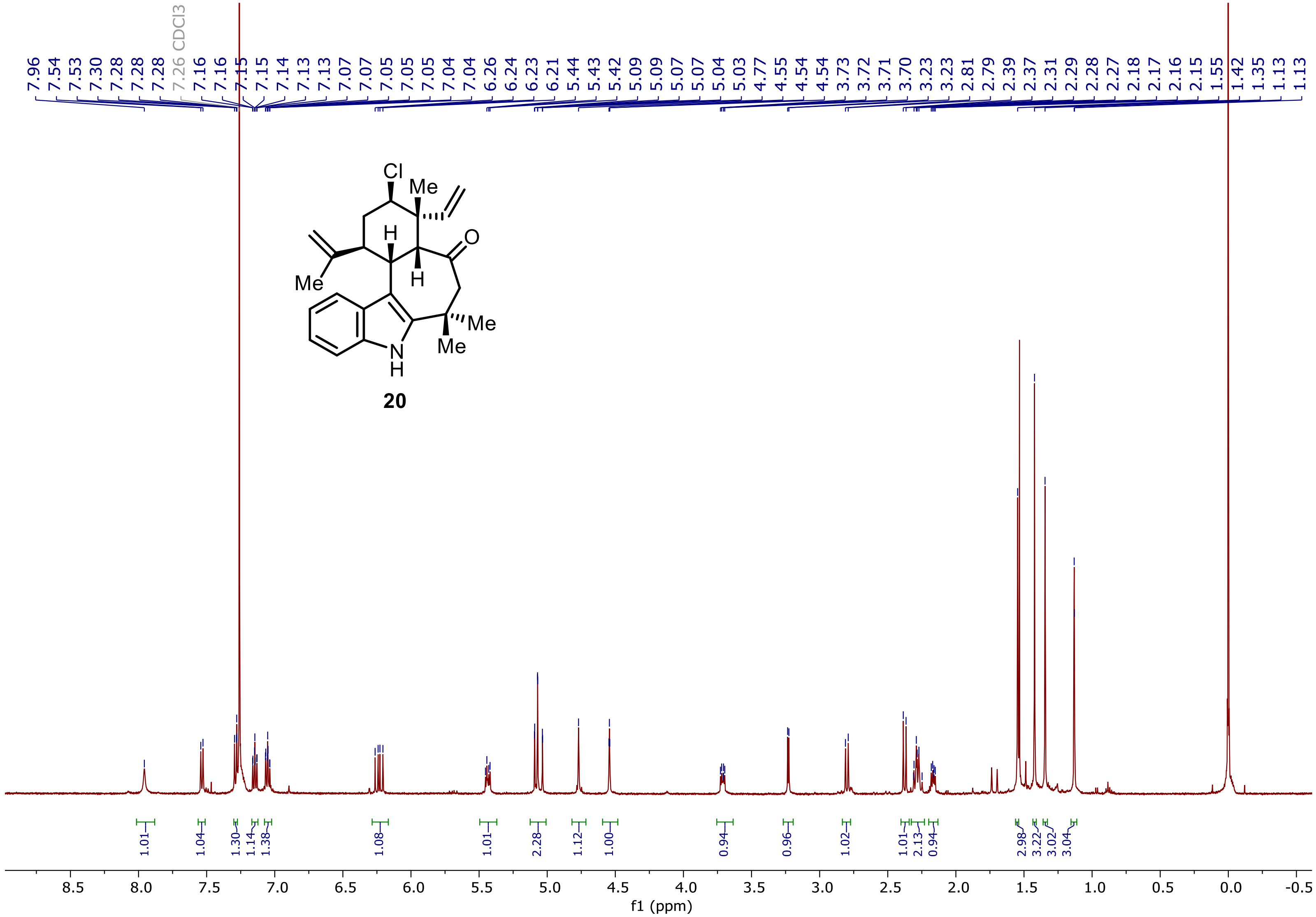

7.96 7.54 7.53 7.30 7.28 7.28 7.28 7.26 CDCl3 7.16 7.16 7.15 7.15 7.14 7.13 7.13 7.07 7.07 7.05 7.05 7.05 7.04 7.04 6.26 6.24 6.23 6.21 5.44 5.43 5.42 5.09 5.09 5.07 5.07 5.04 5.03 4.77 4.55 4.54 4.54 3.73 3.72 3.71 3.70 3.23 3.23 2.81 2.79 2.39 2.37 2.31 2.29 2.28 2.27 2.18 2.17 2.16 2.15 1.55 1.42 1.35 1.13 1.13
Cl
Me
O
H
H
Me
Me
Me
N
H
1.01 1.04 1.30 1.14 1.38 1.08 1.01 2.28 1.12 1.00 0.94 0.96 1.02 1.01 2.13 0.94 2.98 3.22 3.02 3.04
8.5 8.0 7.5 7.0 6.5 6.0 5.5 5.0 4.5 4.0 3.5 3.0 2.5 2.0 1.5 1.0 0.5 0.0 -0.5
f1 (ppm)

20

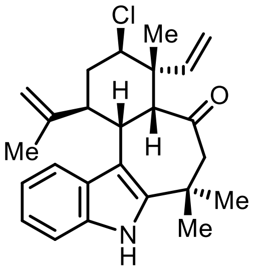
Cl
Me
H
O
Me
H
Me
N
H
Me

20

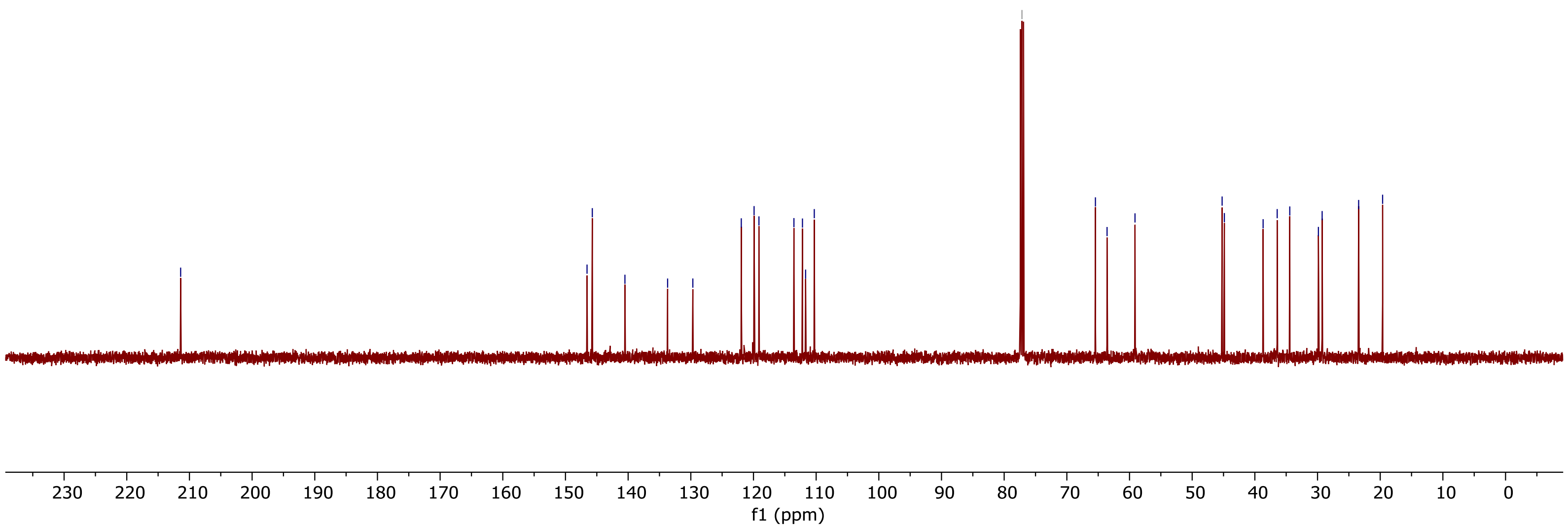
211.4
146.6
145.7
140.5
133.7
129.7
121.9
119.9
119.1
113.5
112.2
111.7
110.3
77.2 CDCl3
65.4
63.6
59.1
45.2
44.9
38.7
36.4
34.4
29.9
29.3
23.4
19.6
230 220 210 200 190 180 170 160 150 140 130 120 110 100 90 80 70 60 50 40 30 20 10 0
f1 (ppm)

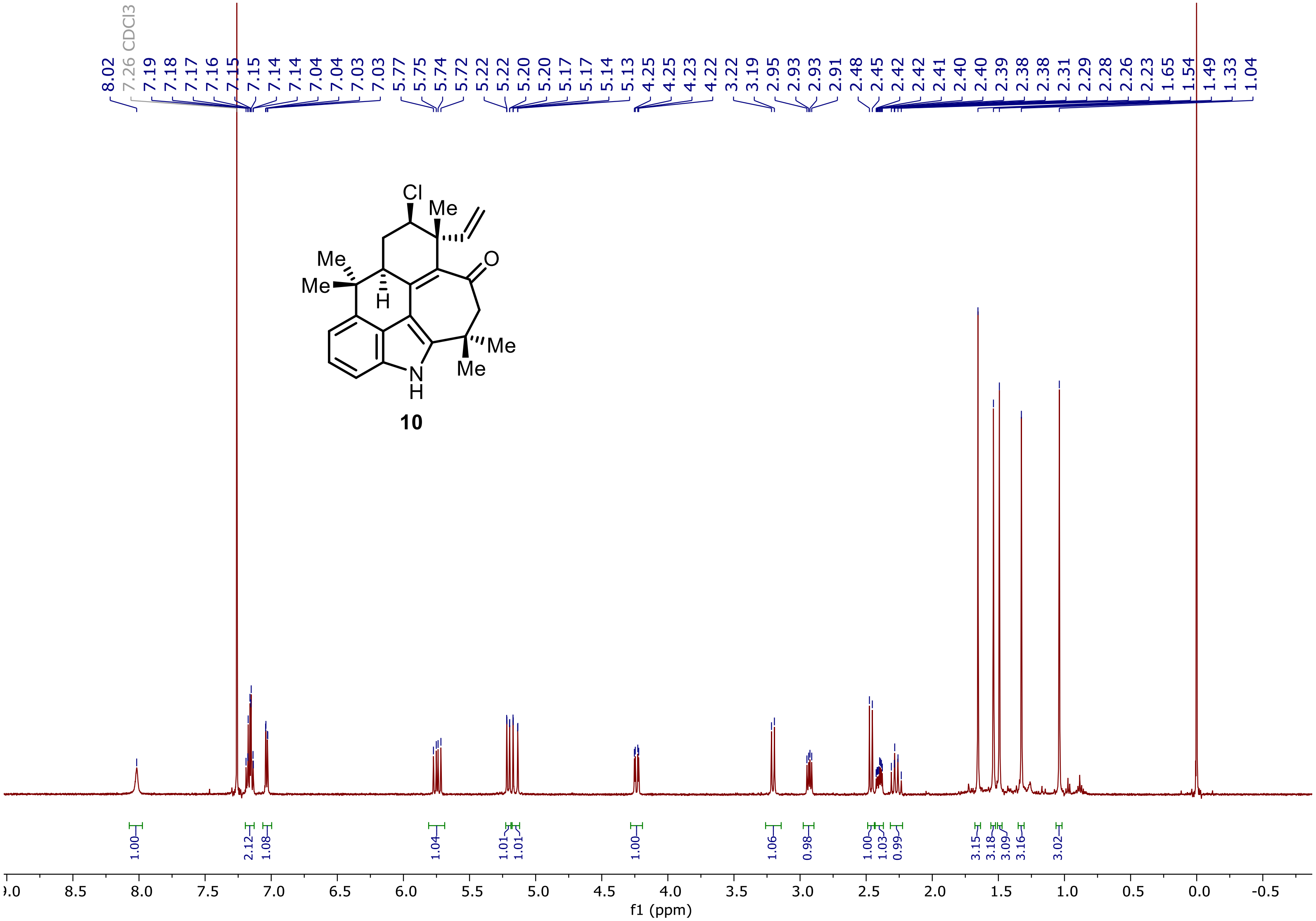
8.02
7.26 CDCl3
7.19
7.18
7.17
7.16
7.15
7.15
7.14
7.14
7.04
7.04
7.03
7.03
5.77
5.75
5.74
5.72
5.22
5.22
5.20
5.20
5.17
5.17
5.14
5.13
4.25
4.25
4.23
4.22
3.22
3.19
2.95
2.93
2.93
2.91
2.48
2.45
2.42
2.42
2.41
2.40
2.40
2.39
2.38
2.38
2.31
2.29
2.28
2.26
2.23
1.65
1.54
1.49
1.33
1.04
Cl
Me
Me
Me
H
O
Me
Me
N
H
1.00
2.12
1.08
1.04
1.01
1.01
1.00
1.06
0.98
1.00
1.03
0.99
3.15
3.18
3.09
3.16
3.02
f1 (ppm)

10

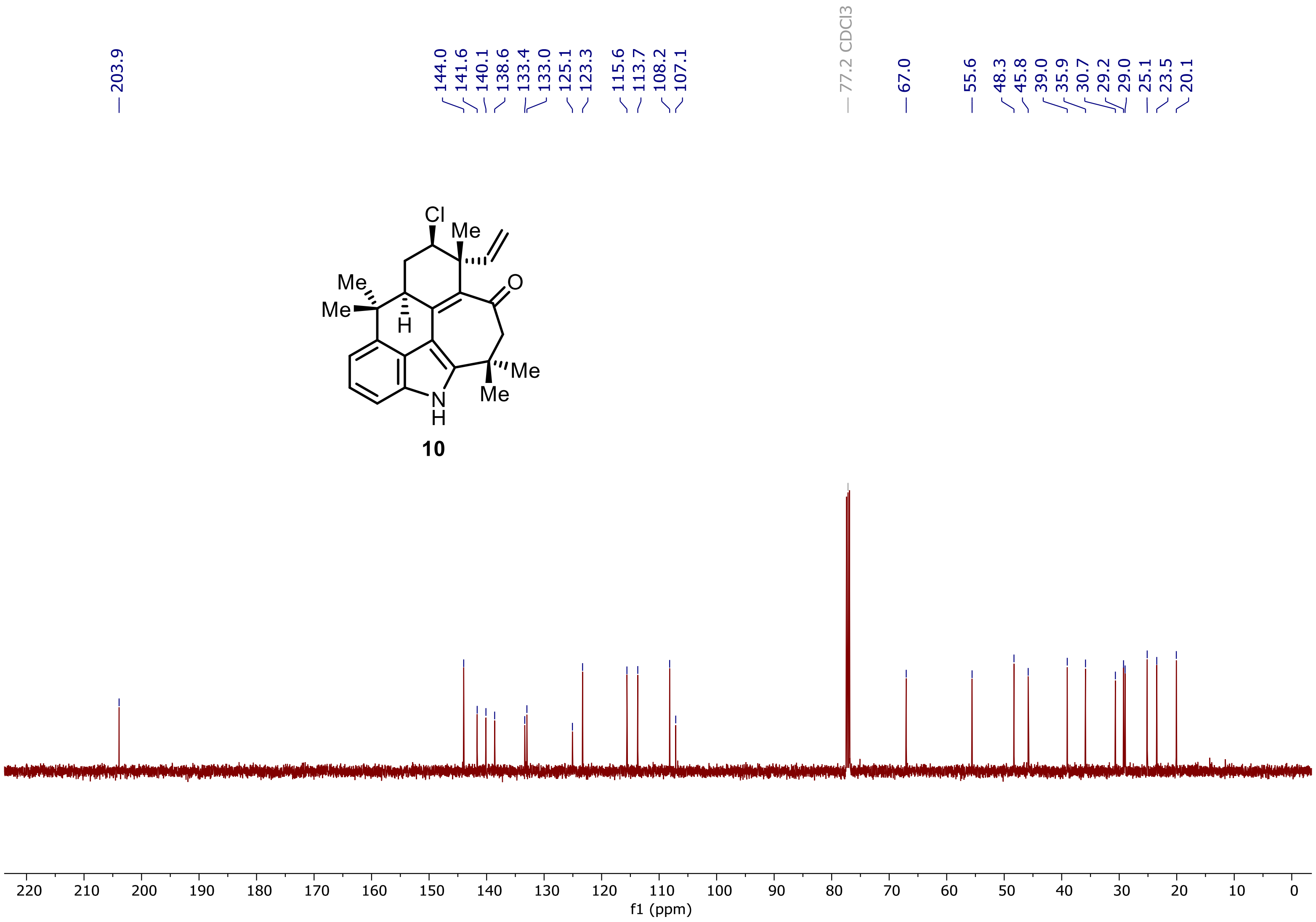

203.9
144.0
141.6
140.1
138.6
133.4
133.0
125.1
123.3
115.6
113.7
108.2
107.1
77.2 CDCl3
67.0
55.6
48.3
45.8
39.0
35.9
30.7
29.2
29.0
25.1
23.5
20.1
Cl
Me
O
Me
Me
H
Me
Me
N
H
10
220 210 200 190 180 170 160 150 140 130 120 110 100 90 80 70 60 50 40 30 20 10 0
f1 (ppm)

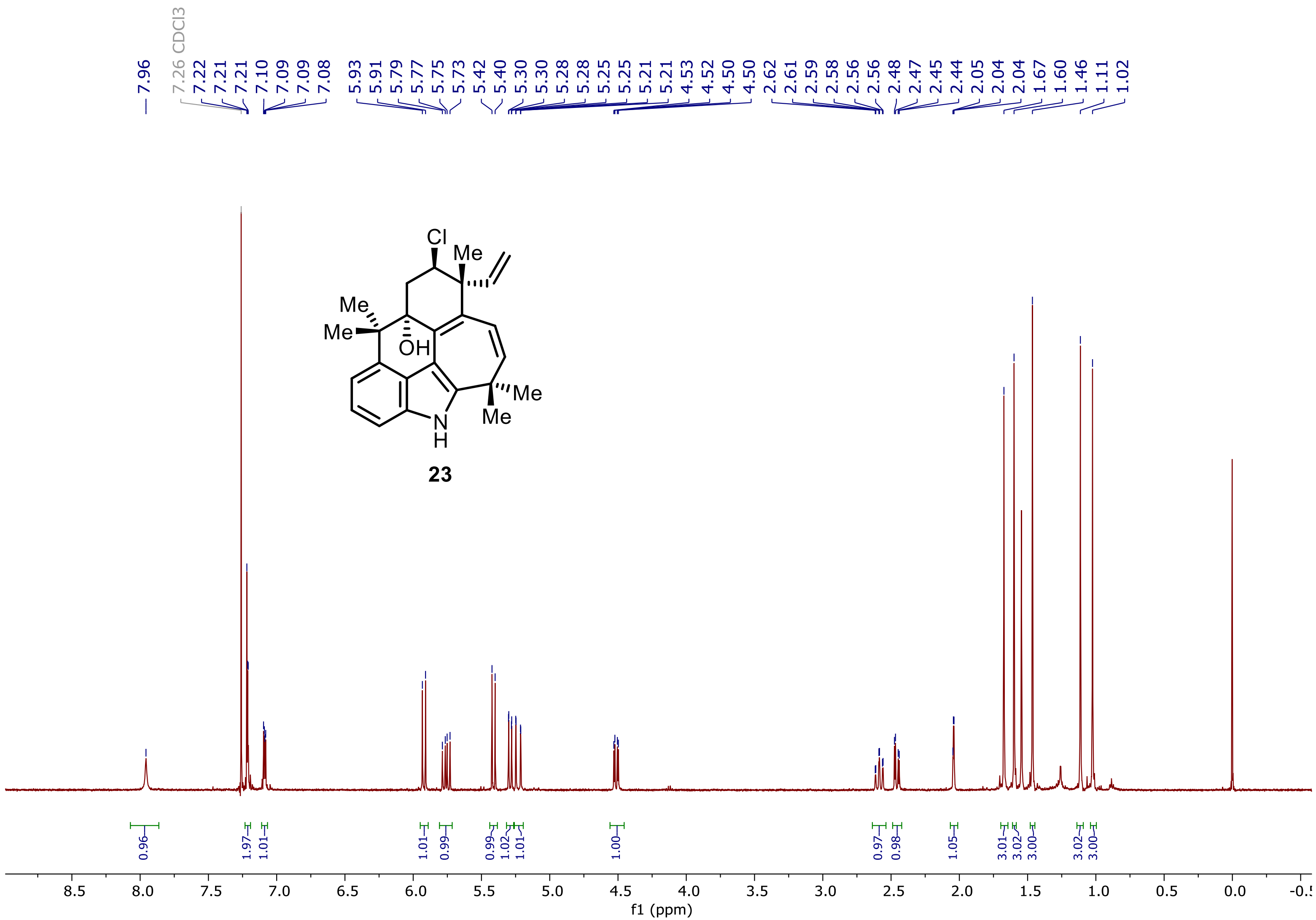
7.96
7.26 CDCl3
7.22
7.21
7.21
7.10
7.09
7.09
7.08
5.93
5.91
5.79
5.77
5.75
5.73
5.42
5.40
5.30
5.30
5.28
5.28
5.25
5.25
5.21
5.21
4.53
4.52
4.50
4.50
2.62
2.61
2.59
2.58
2.56
2.56
2.48
2.47
2.45
2.44
2.05
2.04
2.04
1.67
1.60
1.46
1.11
1.02
Cl
Me
Me
Me
OH
Me
Me
N
H
23
0.96
1.97
1.01
1.01
0.99
0.99
1.02
1.01
1.00
0.97
0.98
1.05
3.01
3.02
3.00
3.02
3.00
8.5
8.0
7.5
7.0
6.5
6.0
5.5
5.0
4.5
4.0
3.5
3.0
2.5
2.0
1.5
1.0
0.5
0.0
f1 (ppm)

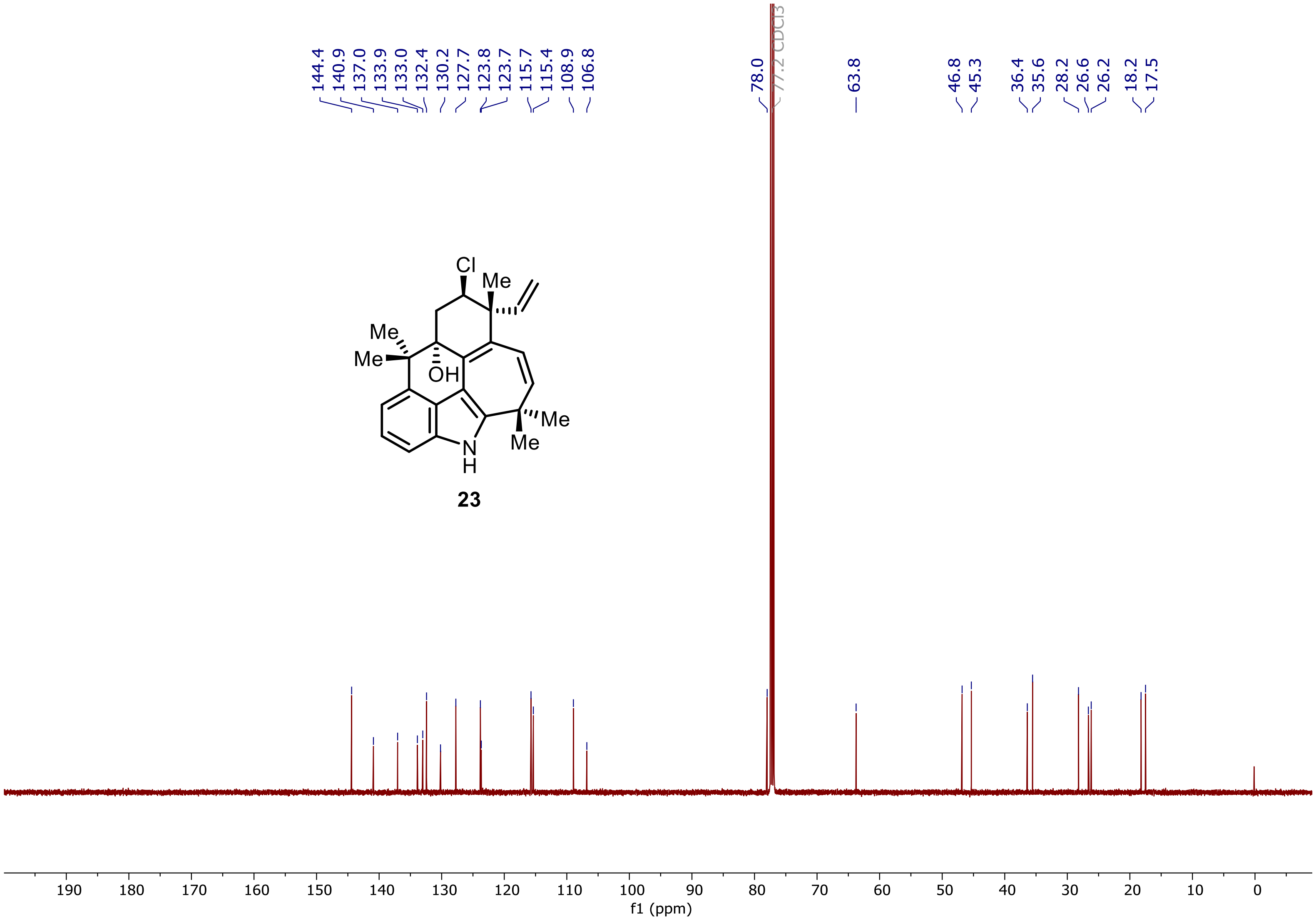

144.4
140.9
137.0
133.9
133.0
132.4
130.2
127.7
123.8
123.7
115.7
115.4
108.9
106.8
78.0
77.2 CDCl3
63.8
46.8
45.3
36.4
35.6
28.2
26.6
26.2
18.2
17.5
Cl
Me
Me
Me
OH
Me
Me
N
H
190 180 170 160 150 140 130 120 110 100 90 80 70 60 50 40 30 20 10 0
f1 (ppm)

23

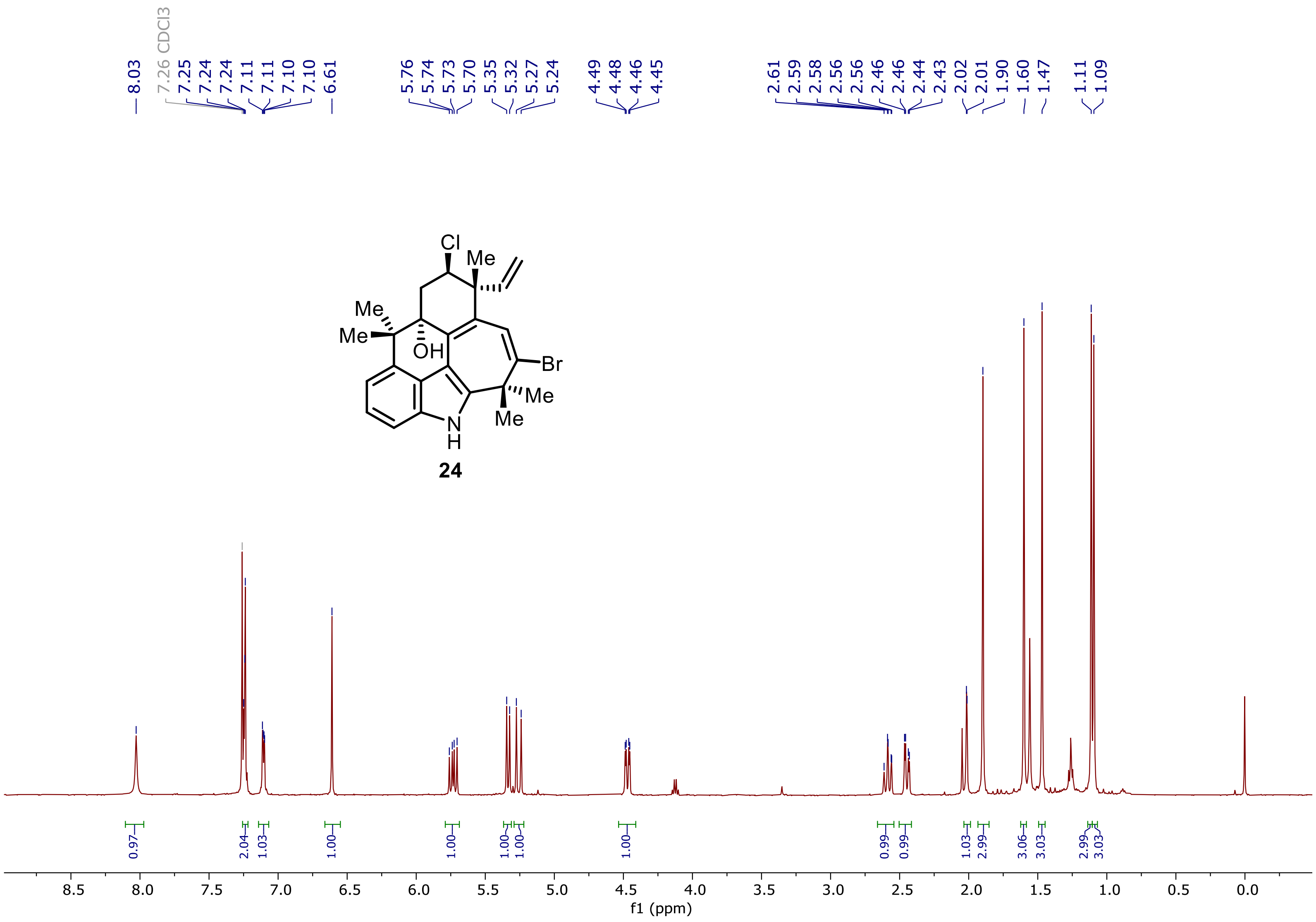
8.03
7.26 CDCl3
7.25
7.24
7.24
7.11
7.11
7.10
7.10
6.61
5.76
5.74
5.73
5.70
5.35
5.32
5.27
5.24
4.49
4.48
4.46
4.45
2.61
2.59
2.58
2.56
2.56
2.46
2.46
2.44
2.43
2.02
2.01
1.90
1.60
1.47
1.11
1.09
Cl
Me
Me
Me
OH
Br
Me
Me
N
H
24
0.97
2.04
1.03
1.00
1.00
1.00
1.00
1.00
0.99
0.99
1.03
2.99
3.06
3.03
2.99
3.03
8.5
8.0
7.5
7.0
6.5
6.0
5.5
5.0
4.5
4.0
3.5
3.0
2.5
2.0
1.5
1.0
0.5
0.0
f1 (ppm)

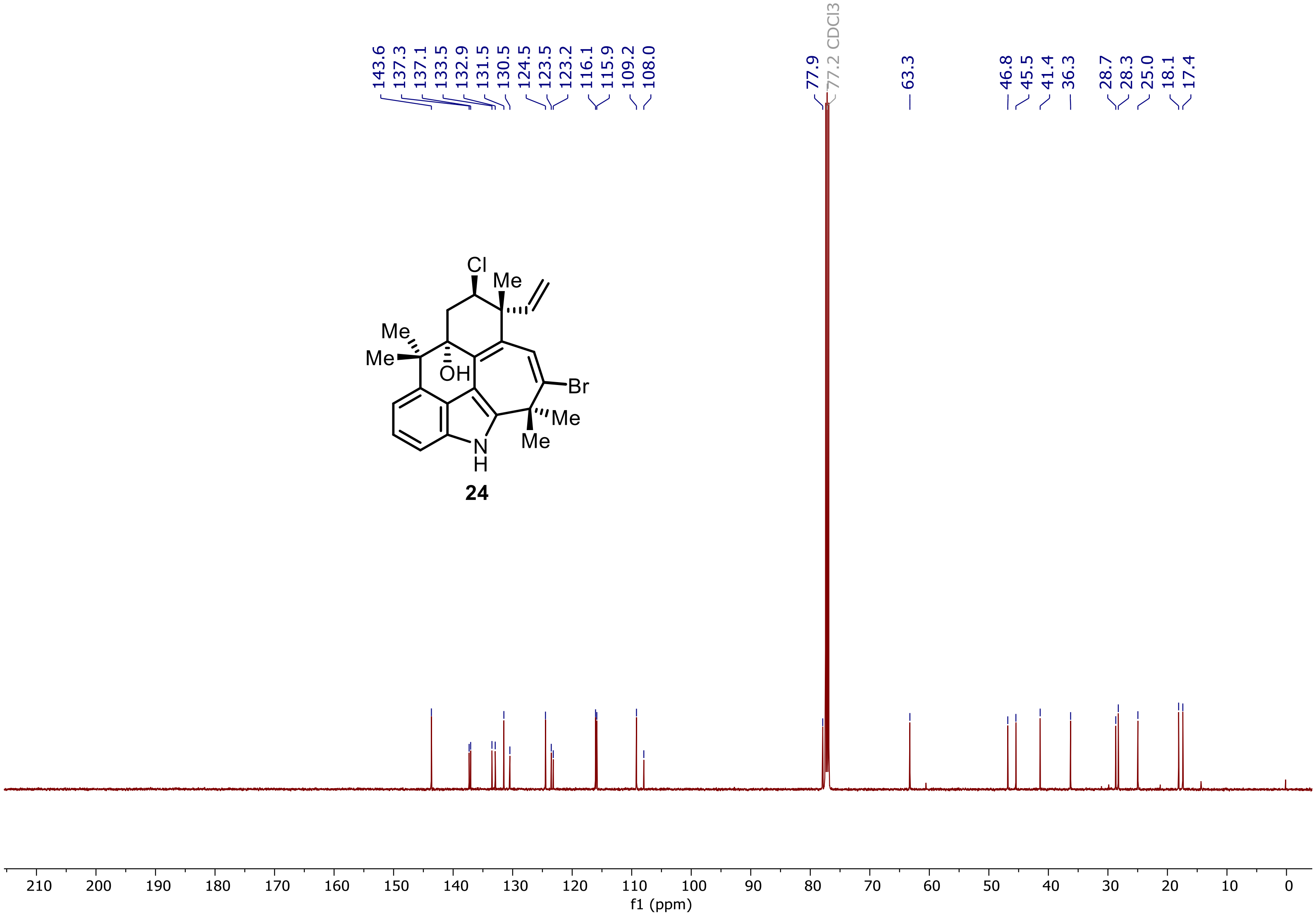
143.6
137.3
137.1
133.5
132.9
131.5
130.5
124.5
123.5
123.2
116.1
115.9
109.2
108.0
77.9
77.2 CDCl3
63.3
46.8
45.5
41.4
36.3
28.7
28.3
25.0
18.1
17.4
Cl
Me
Me
Me
OH
Br
Me
Me
N
H
24
210
200
190
180
170
160
150
140
130
120
110
100
90
80
70
60
50
40
30
20
10
0
f1 (ppm)

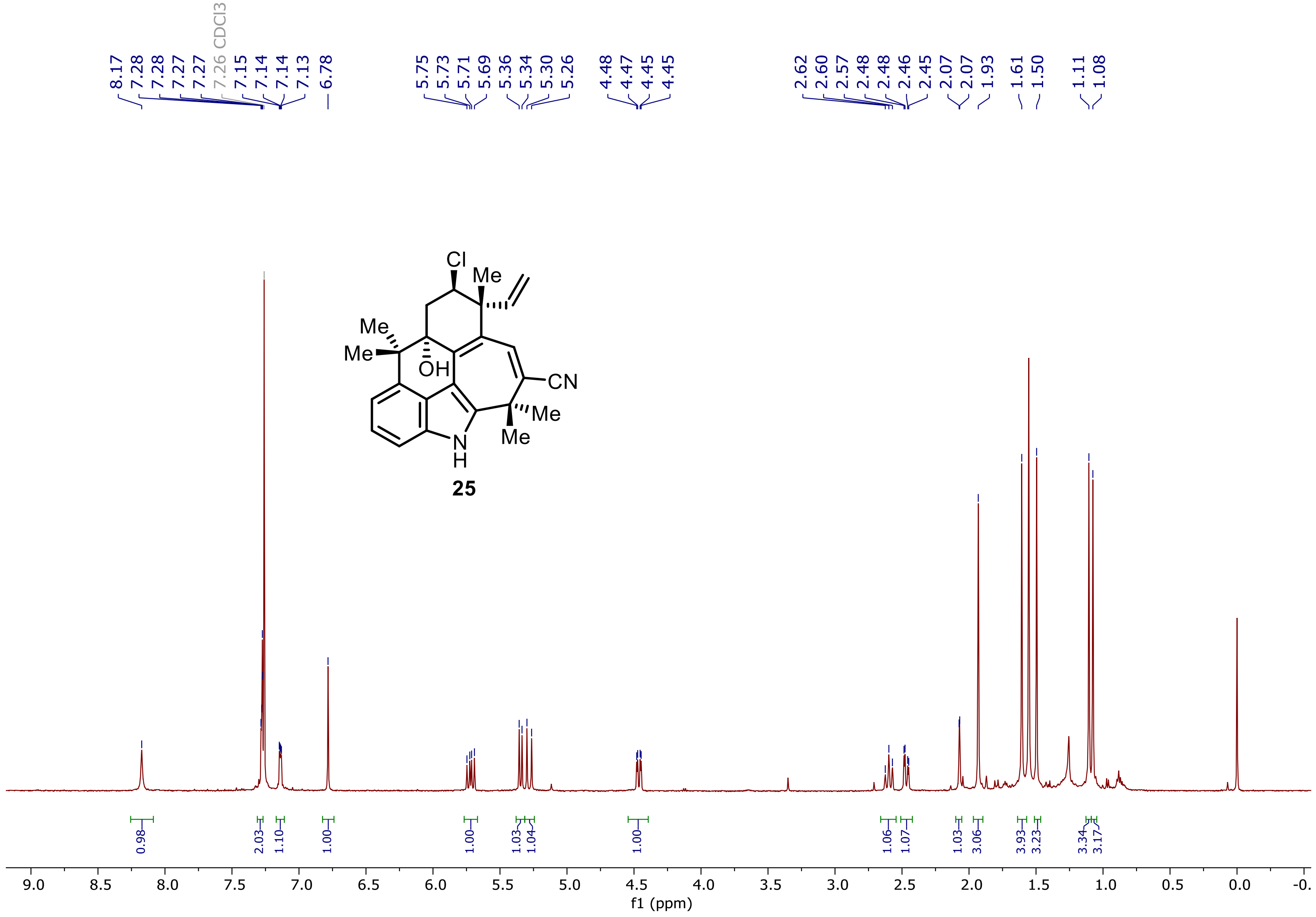
8.17
7.28
7.28
7.27
7.27
7.26 CDCl3
7.15
7.14
7.14
7.13
6.78
5.75
5.73
5.71
5.69
5.36
5.34
5.30
5.26
4.48
4.47
4.45
4.45
2.62
2.60
2.57
2.48
2.48
2.46
2.45
2.07
2.07
1.93
1.61
1.50
1.11
1.08
Cl
Me
Me
Me
OH
CN
Me
Me
N
H
25
0.98
2.03
1.10
1.00
1.00
1.03
1.04
1.00
1.06
1.07
1.03
3.06
3.93
3.23
3.34
3.17
9.0
8.5
8.0
7.5
7.0
6.5
6.0
5.5
5.0
4.5
4.0
3.5
3.0
2.5
2.0
1.5
1.0
0.5
0.0
-0.
f1 (ppm)

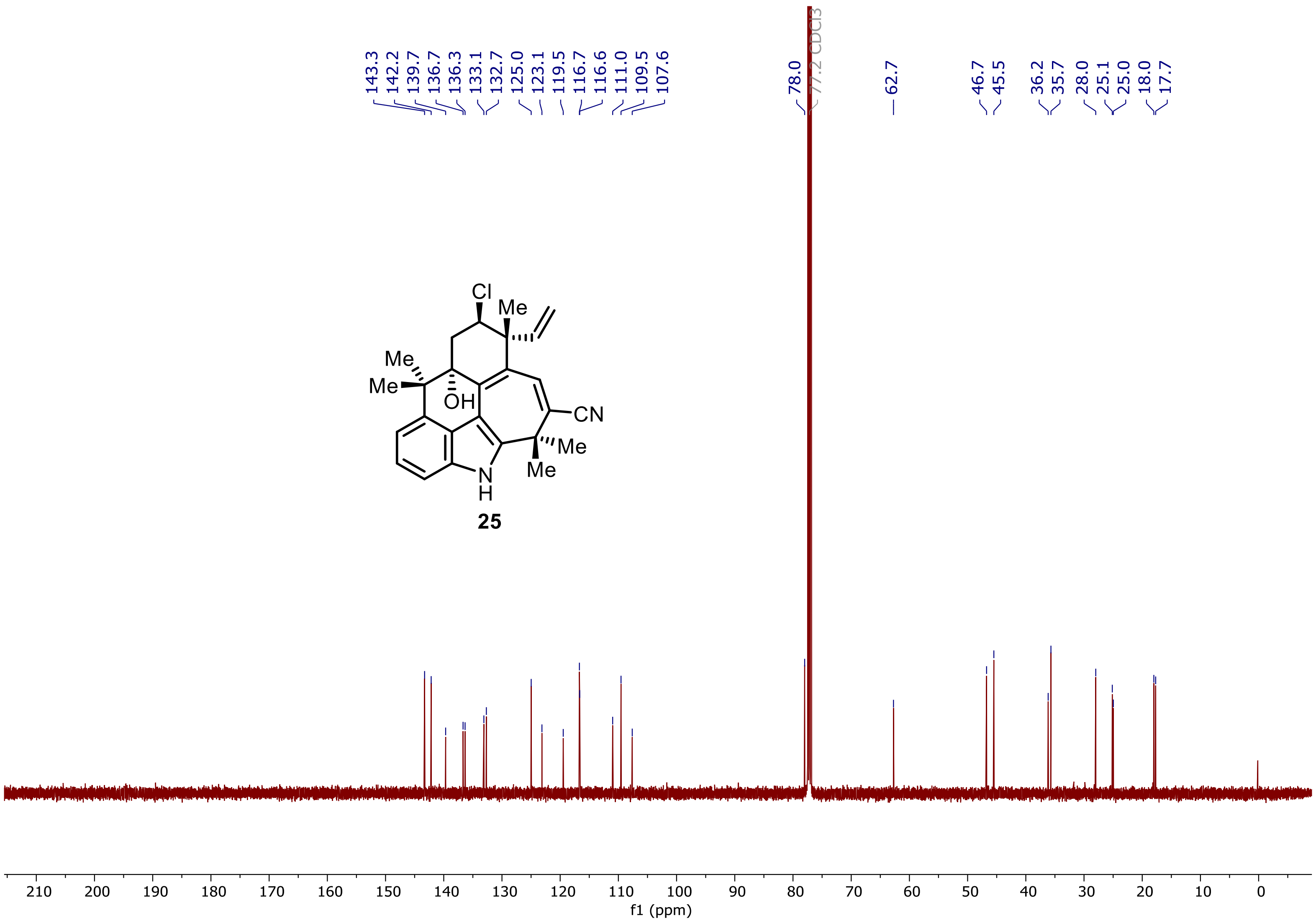
143.3
142.2
139.7
136.7
136.3
133.1
132.7
125.0
123.1
119.5
116.7
116.6
111.0
109.5
107.6
78.0
77.2 CDCl3
62.7
46.7
45.5
36.2
35.7
28.0
25.1
25.0
18.0
17.7
Cl
Me
Me
Me
OH
CN
Me
Me
N
H
25
f1 (ppm)

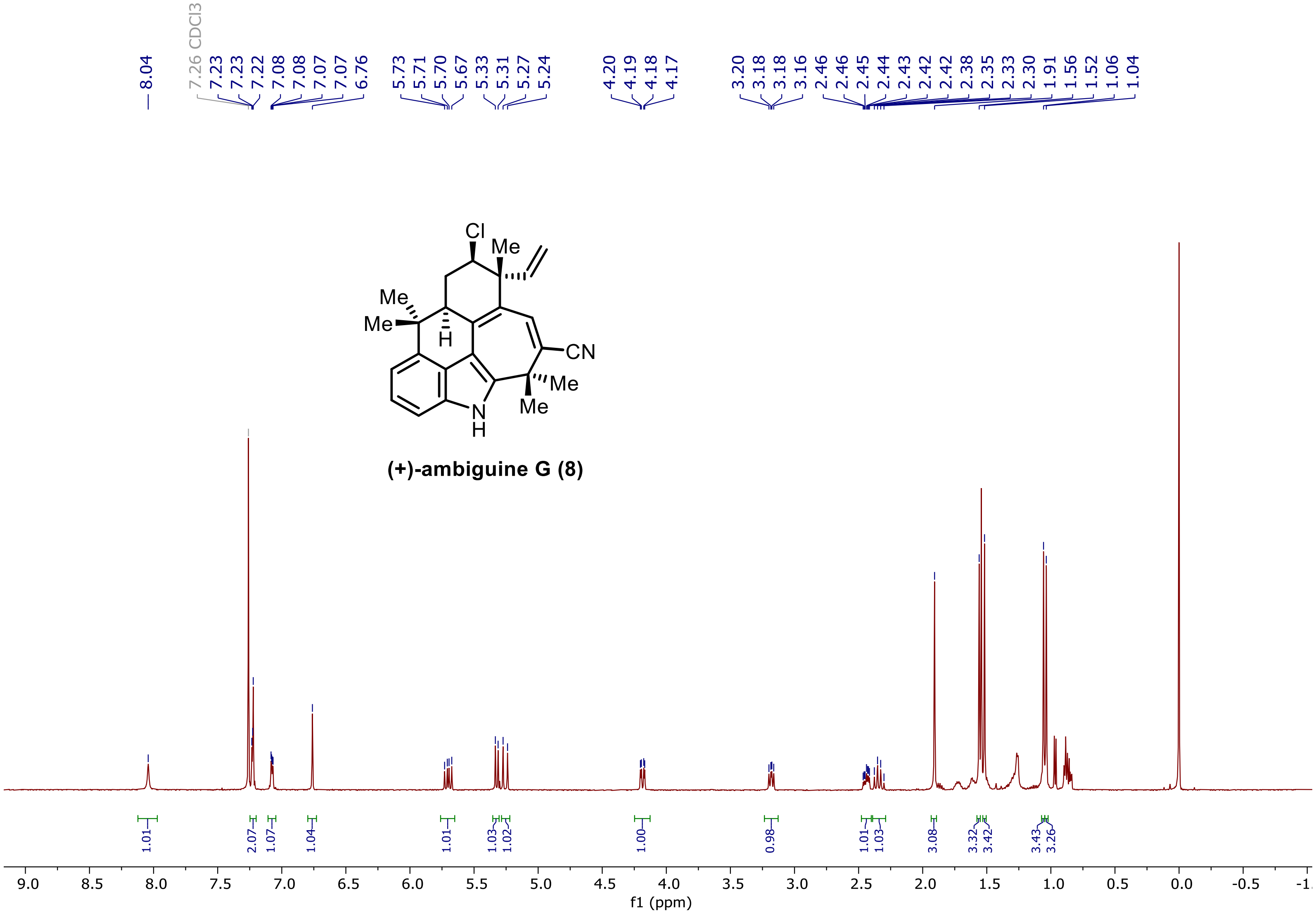
8.04
7.26 CDCl3
7.23
7.23
7.22
7.08
7.08
7.07
7.07
6.76
5.73
5.71
5.70
5.67
5.33
5.31
5.27
5.24
4.20
4.19
4.18
4.17
3.20
3.18
3.18
3.16
2.46
2.46
2.45
2.44
2.43
2.42
2.42
2.38
2.35
2.33
2.30
1.91
1.56
1.52
1.06
1.04
Cl
Me
Me
Me
H
CN
Me
Me
N
H
(+)-ambiguine G (8)
1.01
2.07
1.07
1.04
1.01
1.03
1.02
1.00
0.98
1.01
1.03
3.08
3.32
3.42
3.43
3.26
f1 (ppm)

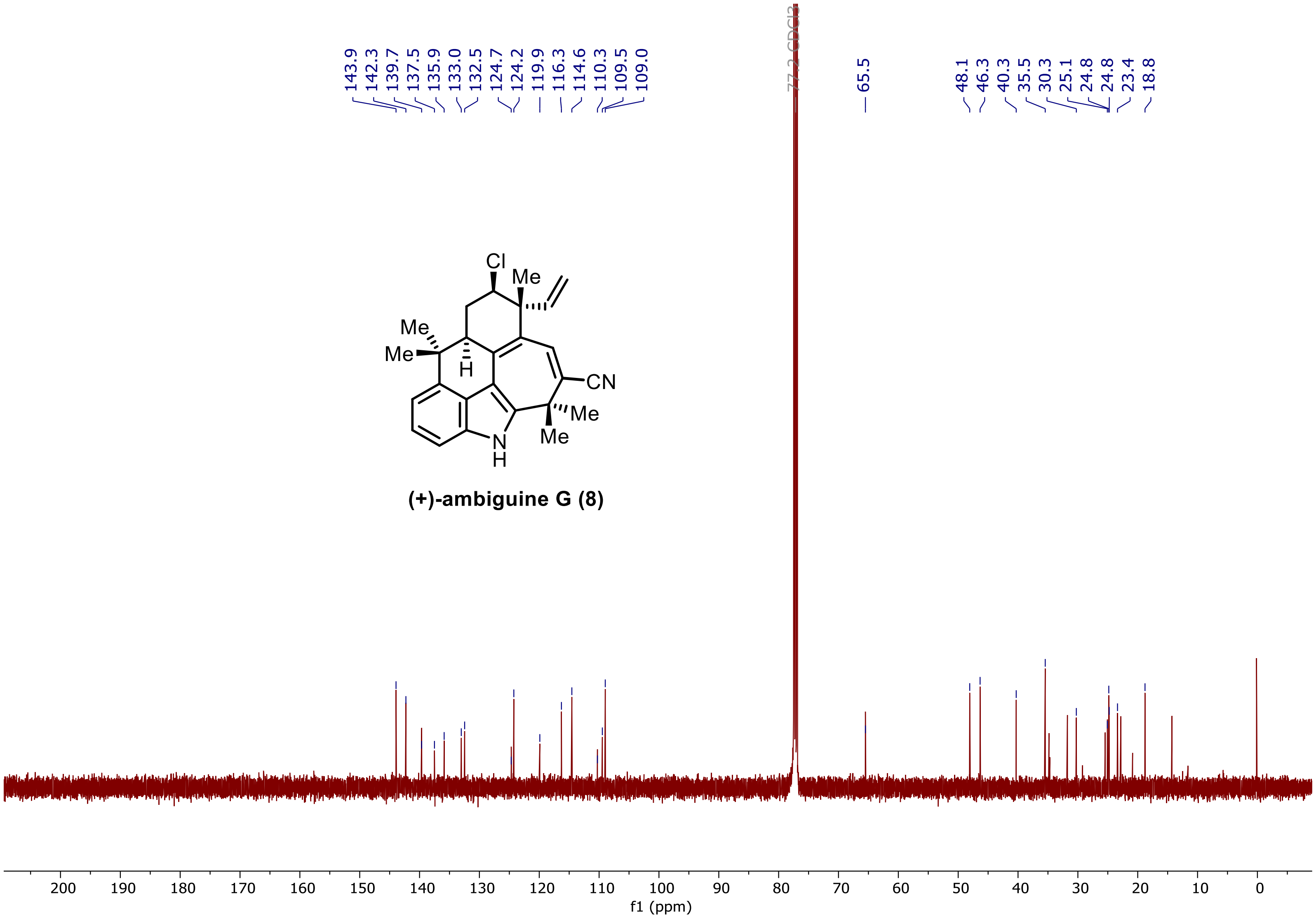

(+)-ambiguine G (8)
